# Live and heat-treated *Lactiplantibacillus plantarum* induce distinct metabolic and immune responses in intestinal epithelial cells

**DOI:** 10.1016/j.isci.2026.115516

**Published:** 2026-04-01

**Authors:** Kaho Matsumoto, Yuta Takada, Yoshiya Imamura, Hina Yoshida, Kazuhiro Sonomura, Mikako Takahashi, Nobuko Moritoki, Tomoko Shindo, Junya Yamamoto, Leonardo Albarracin, Wakako Ikeda-Ohtsubo, Masatoshi Hori, Julio Villena, Fu Namai, Yuji Tsujikawa, Toyoyuki Hashimoto, Keita Nishiyama, Haruki Kitazawa

**Affiliations:** 1 Laboratory of Animal Food Function, Graduate School of Agricultural Science, Tohoku University, Sendai 980-8572, Japan; 2 Laboratory of Applied Entomology, Graduate School of Agricultural Science, Tohoku University, Sendai 980-8572, Japan; 3 Biosystem Diversity Research Group, Molecular Biosystems Research Institute, National Institute of Advanced Industrial Science and Technology (AIST), Tsukuba, Ibaraki 305-8566, Japan; 4 Technology Research Laboratory, Shimadzu Corporation, Nakagyo-ku, Kyoto 604-8511, Japan; 5 Electron Microscope Laboratory, Keio University School of Medicine, Shinjuku-ku, Tokyo 160-8582, Japan; 6 Central Research Institute, ITOEN, Ltd., 21, Mekami, Makinohara-shi, Shizuoka 421-0516, Japan; 7 Laboratory of Immunobiotechnology, Reference Centre for Lactobacilli (CERELA-CONICET), Tucuman 4000, Argentina; 8 Laboratory of Respiratory Immunology (LaRI), Division of Animal Immunology and Omics, CFAI, Graduate School of Agricultural Science, Tohoku University, Sendai 980-8572, Japan; 9 Livestock Immunology Unit, CFAI, Graduate School of Agricultural Science, Tohoku University, Sendai 980-8572, Japan

**Keywords:** immunology, microbiology

## Abstract

Probiotics and postbiotics are distinguished by bacterial viability, a factor that fundamentally influences their interactions with the host. Despite this distinction, how bacterial viability shapes host responses remains unclear because direct comparisons using identical strains are technically challenging. Here, we systematically compared the responses induced by live and heat-treated *Lactiplantibacillus plantarum* 1149^T^ using a microfluidic co-culture system with swine intestinal epithelial cells. Transcriptomics and metabolomics revealed fundamentally distinct epithelial responses depending on bacterial viability. Live L. *plantarum* induced a hypoxia-associated glycolytic shift and PPARG-related transcriptional responses independently of HIF1A, accompanied by the production of lipid mediators including 12,13-diHOME and 9,10-diHOME. In contrast, heat-treated *L. plantarum* induced nuclear factor kappa B (NFKB)-associated pro-inflammatory gene expression in a Toll-like receptor 4 gene (*TLR4)*-dependent manner, likely in response to alterations in bacterial surface components. Our findings demonstrate that bacterial viability influences host epithelial metabolic and immune responses, providing a mechanistic basis for the rational selection of probiotic or postbiotic strategies.

## Highlights

• Compared host responses to live and heat-treated *L. plantarum* using microfluidics
• Live *L. plantarum* induces glycolytic shift and PPARG signaling in epithelia
• Heat-treated *L. plantarum* activates NFKB signaling via altered surface structures
• Bacterial viability shaped epithelial metabolic and immune responses in co-culture

## Graphical abstract

**Figure:**
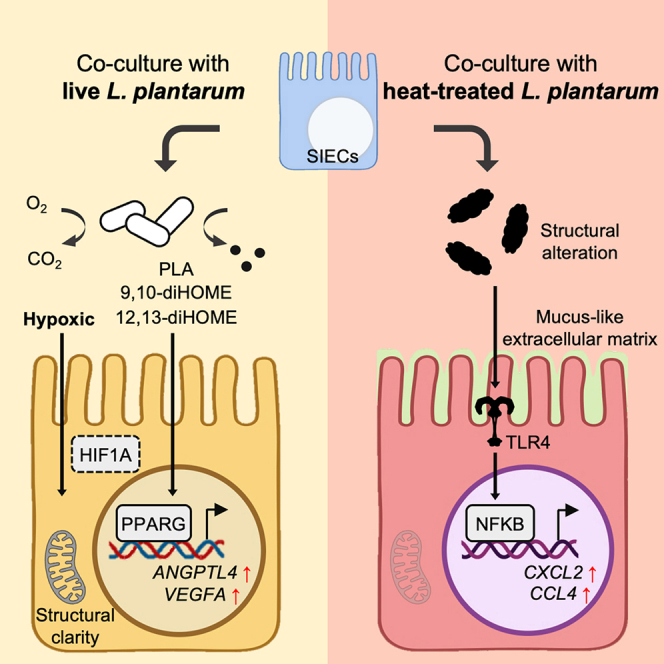

## Introduction

The gut microbiota plays a central role in regulating host immunity, metabolism, and disease susceptibility.^1^^,^^2^ Probiotics, defined as “live microorganisms that confer health benefits when administered in adequate amounts,” help maintain intestinal homeostasis and modulate immune responses.^3^^,^^4^ With the global population aging, the demand for strategies to maintain gut health continues to grow.^5^ Species within Lactobacillaceae and *Bifidobacterium* have been extensively studied for their beneficial effects in mammals, attributed to their metabolic activities and immune-modulatory interactions.^4^^,^^6^ However, the efficacy of probiotics critically depends on bacterial viability, which can be compromised by various abiotic and host-derived stressors, such as temperature, oxygen, pH fluctuations, gastric acid, and bile salts.^7^^,^^8^^,^^9^

Postbiotics, including non-viable microbial cells and/or their components that retain health-promoting properties, can overcome these limitations.^10^ Clinical studies have reported the beneficial effects of heat-inactivated strains, such as *Bifidobacterium bifidum* MIMBb75 and *Lactobacillus acidophilus* LB, in alleviating gastrointestinal disorders.^11^^,^^12^ Consistent with these findings, heat-treated *Lactiplantibacillus plantarum* strains MPL16 and CRL1506 enhance antiviral immunity beyond the gut by strengthening respiratory epithelial cell resistance to SARS-CoV-2.^13^

The biological activities of probiotics and postbiotics are mediated by structural components such as pili,^14^^,^^15^^,^^16^ lipoteichoic acids,^17^^,^^18^ exopolysaccharides,^19^ and microbial nucleic acids.^20^ These molecules engage host pattern recognition receptors and activate intracellular signaling pathways, such as nuclear factor kappa B (NFKB).^21^ In addition, microbial metabolites play a critical role in modulating host physiology. For example, aromatic compounds, including indole-3-lactic acid^22^^,^^23^ and short-chain fatty acids (SCFAs) such as butyrate and propionate,^24^^,^^25^ contribute to immune regulation and intestinal barrier integrity. These metabolites exert their effects in part through the activation of nuclear receptors, including the aryl hydrocarbon receptor and peroxisome proliferator-activated receptor gamma (PPARG), which are key modulators of immune responses and epithelial barrier function.^22^^,^^24^^,^^25^ Despite the growing interest in postbiotics, direct comparisons of the effects of viable versus non-viable bacteria in modulating host responses remain limited. Processing methods, such as heat treatment, profoundly alter bacterial surface properties, thereby influencing host recognition and downstream effects. For instance, spray-drying affects pili abundance in *Lacticaseibacillus rhamnosus* GG,^26^ and thermal treatment modulates the immunogenicity of *Akkermansia muciniphila.*^27^ These observations highlight the need for a systematic investigation of viability-dependent host-microbe interactions.

Bacterial-epithelial co-culture studies have historically been limited by technical challenges, primarily bacterial overgrowth and mismatched oxygen requirements.^28^^,^^29^ Advances in microfluidic systems have enabled precise control of microbial-epithelial co-cultures under physiologically relevant conditions, preserving epithelial integrity while preventing bacterial overproliferation.^30^ In the present study, we employed a microfluidic co-culture platform^31^ (Shimadzu, Kyoto, Japan) to systematically investigate the differential responses of swine intestinal epithelial cells (SIECs) by live and heat-treated *L. plantarum* JCM 1149^T^. Our aim was to elucidate the bacterial molecules and host signaling pathways that mediate viability-dependent metabolic and immunological responses, addressing key gaps in the mechanistic understanding of probiotic and postbiotic functionality.

## Results

### Response of SIECs to live and heat-treated *L. plantarum*

**Figure 1:**
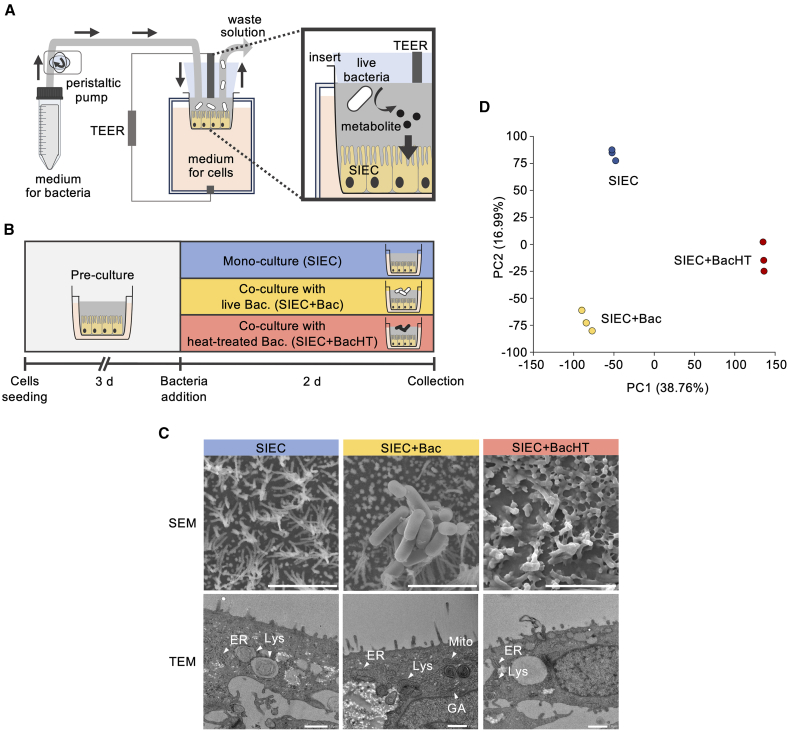
Experimental setup and distinct epithelial responses to live and heat-treated *L. plantarum* (A) Schematic diagram of the microfluidic co-culture system enabling controlled interactions between swine intestinal epithelial cells (SIECs) and live or heat-treated *Lactiplantibacillus plantarum* JCM 1149^T^ under continuous medium flow or static conditions. (B) Experimental timeline outlining cell seeding, bacterial addition (live or heat-treated), and sample collection after 48 h of incubation. (C) Representative scanning electron microscopy (SEM) and transmission electron microscopy (TEM) images illustrating morphological differences among SIEC (monoculture), SIEC+Bac (live bacteria co-culture), and SIEC+BacHT (heat-treated bacteria co-culture) conditions. TEM images showing cellular organelles, including endoplasmic reticulum (ER), lysosomes (Lys), mitochondria (Mito), and Golgi apparatus (GA). Scale bars: SEM, 3 μm; TEM, 1 μm. SEM and TEM analyses were each performed once per condition. A total of 30, 28, and 24 SEM fields and 19, 18, and 67 TEM fields were randomly acquired for SIEC, SIEC+Bac, and SIEC+BacHT, respectively. Additional images are provided in Figures S2 and S3. (D) Principal component analysis of RNA sequencing data highlights distinct transcriptional profiles induced depending on the bacterial viability state. Data are mean values from three independent biological replicates (*n* = 3).

*L. plantarum* is commonly found in fermented foods and is widely recognized for its beneficial roles in mammalian gut health.^32^^,^^33^ To investigate how bacterial viability influences host epithelial responses, we co-cultured SIECs with live or heat-treated *L. plantarum* JCM 1149^T^ using a microfluidic co-culture system (Figures 1A and S1A). Specifically, live bacterial cells were cultured with SIECs under continuous medium flow in diluted modified Gifu anaerobic broth (mGAM) medium for 48 h (SIEC+Bac), whereas heat-treated bacterial cells were cultured statically for the same duration (SIEC+BacHT) (Figure 1B). The bacterial suspension was diluted to either 2.8 × 10^5^ colony-forming unit (CFU)/mL (OD_600_ = 0.001) or 4.0 × 10^6^ (CFU)/mL (OD_600_ = 0.01) for inoculation, and bacterial growth in SIEC+Bac was confirmed after 48 h (Figure S1B). At the lower inoculation level, bacterial growth was insufficient for RNA sequencing (Figure S1B). In addition, transepithelial electrical resistance (TEER) was stable in monocultured SIECs (SIEC) and SIEC+Bac at the higher inoculation level (Figure S1C). These results indicated that exposure to live bacteria did not exert detrimental effects when inoculated at 4.0 × 10^6^ CFU/mL (OD_600_ = 0.01). The pH of the medium remained stable across the SIEC, SIEC+Bac, and SIEC+BacHT conditions (Table S1), excluding acidification as a confounding factor.

To investigate qualitative structural differences between live and heat-treated bacteria, we performed scanning electron microscopy (SEM) and transmission electron microscopy (TEM). SEM revealed morphological differences associated with bacterial viability. Intact microvilli and preserved epithelial surface structures were observed in both the SIEC and SIEC+Bac conditions, with bacterial adhesion detected in SIEC+Bac (Figures 1C and S2). In contrast, SIEC+BacHT exhibited a mucus-like extracellular matrix on the epithelial surface, suggesting a host-specific response to heat-treated bacteria (Figures 1C and S2). TEM revealed that SIEC+Bac exhibited structural clarity of the lipid membranes of the endoplasmic reticulum, lysosomes, mitochondria, and Golgi apparatus as compared with SIEC and SIEC+BacHT (Figures 1C and S3).

To elucidate transcriptional responses associated with bacterial viability, SIECs cultured under the SIEC, SIEC+Bac, and SIEC+BacHT conditions were subjected to RNA sequencing. Principal component analysis revealed a clear separation among the three conditions, underscoring the distinct transcriptomic profiles induced by live and heat-treated bacteria (Figure 1D). These results collectively demonstrate that viable and non-viable *L. plantarum* induce unique transcriptional programs in epithelial cells.

### Live *L.plantarum* induces hypoxia and glycolysis

**Figure 2:**
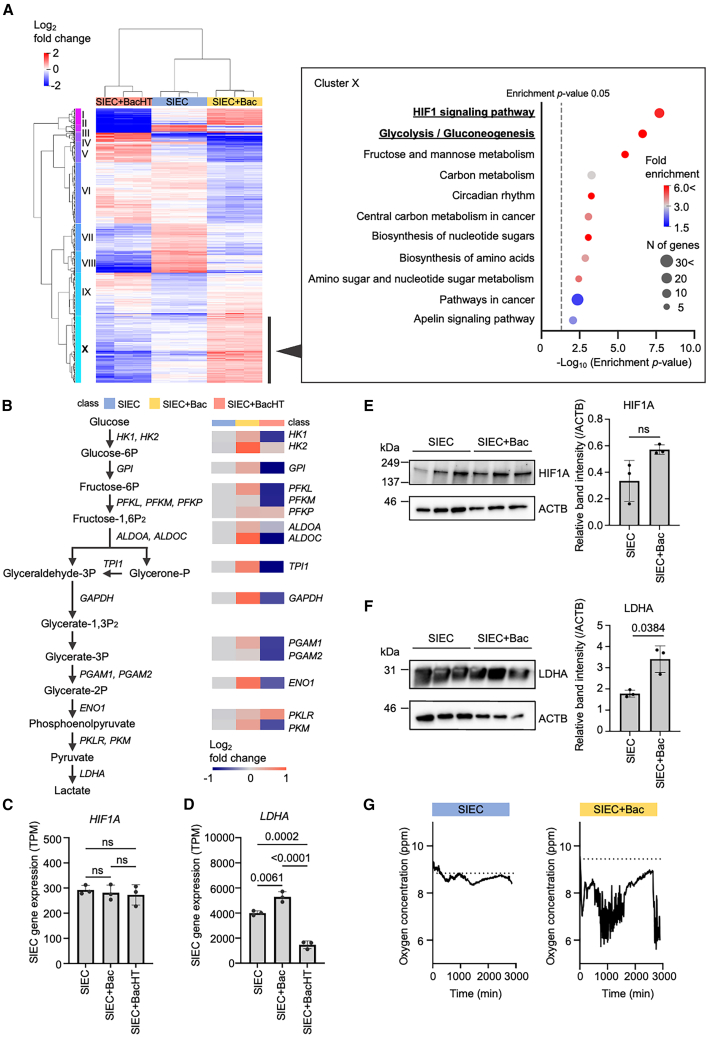
Hypoxia response and enhanced glycolysis in SIECs induced by live *L. plantarum* co-culture (A) Cluster map illustrating significantly differentially expressed genes (DEGs, adjusted *p* < 0.001) between monocultured SIEC and SIEC+Bac (*n* = 3). Top enriched functional pathways among upregulated genes are indicated on the right (Enrichment *p* value <0.001). (B) Diagram of the glycolytic pathway showing changes in gene expression (log_2_ fold change) for key enzymes in SIECs under SIEC+Bac conditions. Metabolite abbreviations: Glucose-6P, glucose-6-phosphate; Fructose-6P, fructose-6-phosphate; Fructose-1,6P2, fructose-1,6-bisphosphate; Glycerone-P, dihydroxyacetone phosphate; Glycerate-1,3P_2_, 1,3-bisphosphoglycerate; Glycerate-3P, 3-phosphoglycerate; Glycerate-2P, 2-phosphoglycerate. (C and D) TPM of HIF1A (C) and LDHA (D) in SIECs. Data are presented as mean ± SD from three independent biological replicates (*n* = 3), each analyzed in technical triplicate. Statistical significance was determined using one-way ANOVA with Tukey’s post-hoc test; *ns*, not significant. (E and F) Western blot analysis of HIF1A (E), LDHA (F), and Actin Beta (ACTB) protein levels in SIECs under the indicated conditions. Band intensities were quantified and normalized to ACTB, and relative protein levels are shown. Data are presented as mean ± SD from three independent biological replicates (*n* = 3). Statistical significance was determined using Welch’s *t* test; ns, not significant. Full, uncropped western blot images are shown in Figures S4C and S4D. (G) Real-time oxygen concentration measured in the culture device. The dotted line on the x axis indicates the initial oxygen concentration. Data shown are from one independent experiment that yielded results comparable to those of other experiments. The remaining data are shown in Figure S4I, and the raw data are provided in Table S3.

We assessed how live *L. plantarum* influences host epithelial transcription by comparing differentially expressed genes (DEGs) between SIEC and SIEC+Bac. In total, 2,688 DEGs were identified (adjusted *p* < 0.001) (Figure S4A). The hypoxia-inducible factor-1 (HIF-1) signaling pathway was significantly enriched among the genes upregulated under co-culture conditions (Figure 2A and Table S2). This upregulated gene cluster, cluster X, also showed marked induction of glycolytic genes, whereas genes involved in mitochondrial metabolism were downregulated (Figures 2B and S4B).

Given this transcriptional signature, we focused on the HIF-1 signaling pathway. Further examination of the this pathway revealed that, despite the pathway enrichment, *HIF1A* transcript levels did not differ between SIEC and SIEC+Bac (Figure 2C). In contrast, several HIF-1-associated metabolic genes were transcriptionally induced under co-culture conditions. Notably, lactate dehydrogenase A (*LDHA*), a target gene of HIF1A and involved in glycolysis,^34^ was strongly upregulated at the mRNA level in SIEC+Bac but suppressed in SIEC+BacHT (Figure 2D). To determine whether these transcriptional changes were reflected at the protein level, we examined HIF1A and LDHA protein abundance by western blot analysis. Consistent with the RNA sequencing data, HIF1A protein levels remained comparable between SIEC and SIEC+Bac, whereas LDHA protein levels were increased in SIEC+Bac (Figures 2E, 2F, S4C, and S4D). These results indicate the induction of hypoxia-associated metabolic programs in SIEC+Bac without a detectable increase in total HIF1A protein abundance. In addition, MAX interactor 1 (*MXI1*), a downstream target of HIF-1 signaling,^35^ was significantly upregulated under SIEC+Bac conditions (Figure S4E). Together, these findings suggest hypoxia-associated metabolic reprogramming that occurs independently of HIF1A activation, potentially involving alternative regulatory pathways such as Myc signaling.^36^^,^^37^ To assess the metabolic consequences of these changes, metabolomic profiling was performed. Lactate levels were specifically elevated in SIEC+Bac (Figure S4F),^38^ indicating enhanced glycolytic flux.

Considering that hypoxia is a key driver of glycolytic metabolism, we examined whether co-culture with live bacteria alters local oxygen availability. Oxygen concentrations at the apical side of the epithelium were continuously monitored using an oxygen measurement sensor (PreSens, Regensburg, Germany) under continuous medium flow (Figures S4G and S4H). Oxygen levels were significantly reduced in SIEC+Bac relative to SIEC (Figures 2G and S4I, and Table S3), confirming the establishment of a hypoxic microenvironment by live bacteria. Collectively, these data support the notion that co-culture with live *L. plantarum* may induce a hypoxia-associated shift toward glycolytic metabolism in SIECs, independent of the activation of HIF1A. Finally, to directly assess the impact of oxygen availability on epithelial metabolism, SIECs were cultured under anaerobic conditions. Western blot analysis revealed no significant differences in HIF1A or LDHA protein levels between aerobic and anaerobic conditions (Figures S5A and S5B). However, lactate levels were significantly elevated under anaerobic conditions (Figure S5C). This finding suggests that lactate accumulation can be driven by metabolic flux and redox regulation rather than changes in LDHA protein abundance alone.

### Heat-treated *L. plantarum* induces immune activation via NFKB signaling

**Figure 3:**
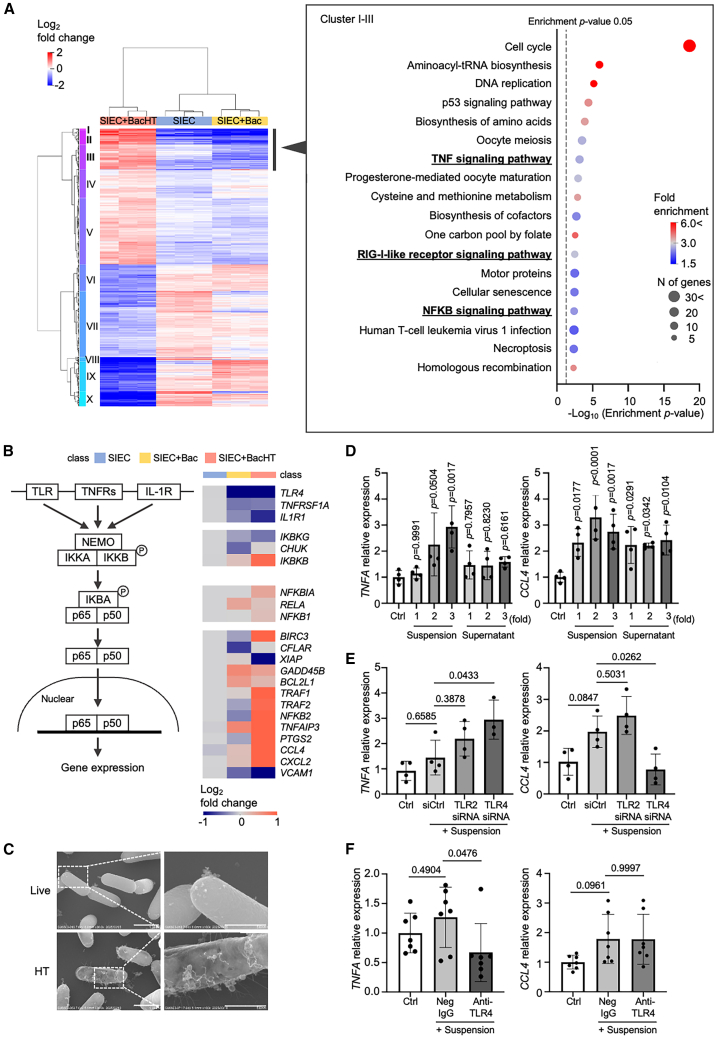
NFKB pathway-mediated immune activation in SIECs triggered by heat-treated *L. plantarum* (A) Cluster map depicting DEGs (adjusted *p* < 0.001) between monocultured SIECs and SIEC+BacHT (*n* = 3). Top enriched pathways among upregulated genes are indicated on the right (Enrichment *p* value <0.005). (B) NFKB signaling pathway schematic highlighting transcriptomic alterations induced by heat-treated bacteria. Key molecules: TLR, Toll-like receptor; TNFRs, tumor necrosis factor receptors; IL-1R, interleukin-1 receptor; NEMO, NFKB essential modulator; IKBA, inhibitor of KB alpha. (C) Representative SEM images of heat-treated bacterial morphology, characterized by damaged cell surface. Scale bars, 1 μm and 500 nm. (D) RT-qPCR analysis of *TNFA* and *CCL4* expression in SIECs treated with diluted BacHT suspension or supernatant. The x axis represents relative doses corresponding to 1×, 2×, and 3× the standard treatment concentration, where higher values indicate proportionally greater amounts of the original BacHT preparation. Data are presented as mean ± SD from four biological replicates (*n* = 4), each measured in technical triplicate. Statistical analysis was performed using one-way ANOVA with Dunnett’s multiple comparisons test. (E) RT-qPCR analysis of *TNFA* and *CCL4* expression in SIECs after TLR2 or TLR4 knockdown (siRNA transfection) followed by stimulation with BacHT. “siCtrl” indicates cells transfected with non-targeting negative control siRNA duplexes. Data are presented as mean ± SD from four biological replicates (*n* = 4), each measured in technical triplicate. Statistical analysis was performed using one-way ANOVA with Dunnett’s multiple comparisons test. (F) RT-qPCR analysis of *TNFA* and *CCL4* expression in SIECs pretreated with a TLR4-neutralizing antibody or isotype control IgG, followed by stimulation with BacHT. “Neg IgG” indicates cells pretreated with isotype control IgG. Data are presented as mean ± SD from seven independent biological replicates (*n* = 7), each measured in technical triplicate. Statistical significance was determined using one-way ANOVA with Sidak’s multiple *t* test.

To assess the effects of heat-treated *L. plantarum* on epithelial gene expression, we compared transcriptomic profiles between SIEC and SIEC+BacHT. In total, 5,192 DEGs were identified (adjusted *p* < 0.001), nearly double the number observed in SIEC+Bac (Figures 3A and S6A). Enrichment analysis of the upregulated genes revealed the activation of immune-related pathways, including tumor necrosis factor (TNF) signaling, retinoic acid-inducible gene I (RIG-I)-like receptor signaling, and the NFKB signaling cascade (Figures 3A, 3B, S6B, and Table S4). As NFKB can be activated downstream of both TNF and RIG-I signaling, we focused on NFKB pathway components and their downstream targets (Figure 3B).^39^^,^^40^ The expression of NFKB signaling molecules, including TNF receptor-associated factor 2 (*TRAF2*) and *NFKB2*, a mediator of the non-canonical NFKB pathway,^41^^,^^42^ was markedly upregulated in SIEC+BacHT. Moreover, the expression of downstream chemokines such as C-C motif chemokine ligand 4 (*CCL4*) and C-X-C motif chemokine ligand 2 (*CXCL2*) was significantly induced, collectively indicating a multifaceted activation of the NFKB signaling axis, distinct from the glycolytic reprogramming observed in response to live bacteria (Figure 3B and Table S4).

The transcriptomic profile of SIEC+BacHT revealed an immune activation pattern distinct from that induced by live bacteria. To investigate potential triggers of the BacHT-mediated immune response, we utilized SEM to examine morphological differences between live and heat-treated bacteria. SEM imaging revealed pronounced surface irregularities and membrane disruptions in heat-treated cells (Figures 3C and S6C), indicating surface damage and alterations in cell surface antigens, potentially leading to the leakage of intracellular components. Heat treatment resulted in a substantial release of nucleic acids (Figure S6D); however, exposure of SIECs to either purified nucleic acid mixture or BacHT-derived supernatants did not induce the expression of *TNFA* or *CCL4*—two representative markers of NFKB activation (Figures 3D and S6E). In contrast, exposure to heat-treated bacterial cells (i.e., a suspension) significantly upregulated both *TNFA* and *CCL4* expression (Figure 3D). Moreover, small interfering (si) RNA-mediated knockdown of Toll-like receptor 4 (*TLR4*) expression significantly reduced *CCL4* expression (Figure 3E), indicating that the observed NFKB activation is mediated, at least in part, through TLR4 signaling. Consistent with this observation, treatment with a TLR4-neutralizing antibody significantly reduced *TNFA* expression induced by heat-treated bacterial suspension (Figure 3F), further supporting the involvement of TLR4 signaling in the BacHT-mediated NFKB response. Together, these findings suggest that NFKB signaling activation induced by heat-treated *L. plantarum* is not triggered by released intracellular components such as nucleic acids but rather by structural alterations of the bacterial cell surface. These results highlight a distinct immunostimulatory mechanism in which processing-dependent modifications to bacterial cell architecture govern host epithelial NFKB responses via TLR4.

### Regulation of aromatic amino acid and lipid biosynthesis in live *L. plantarum* co-cultured with SIECs

**Figure 4:**
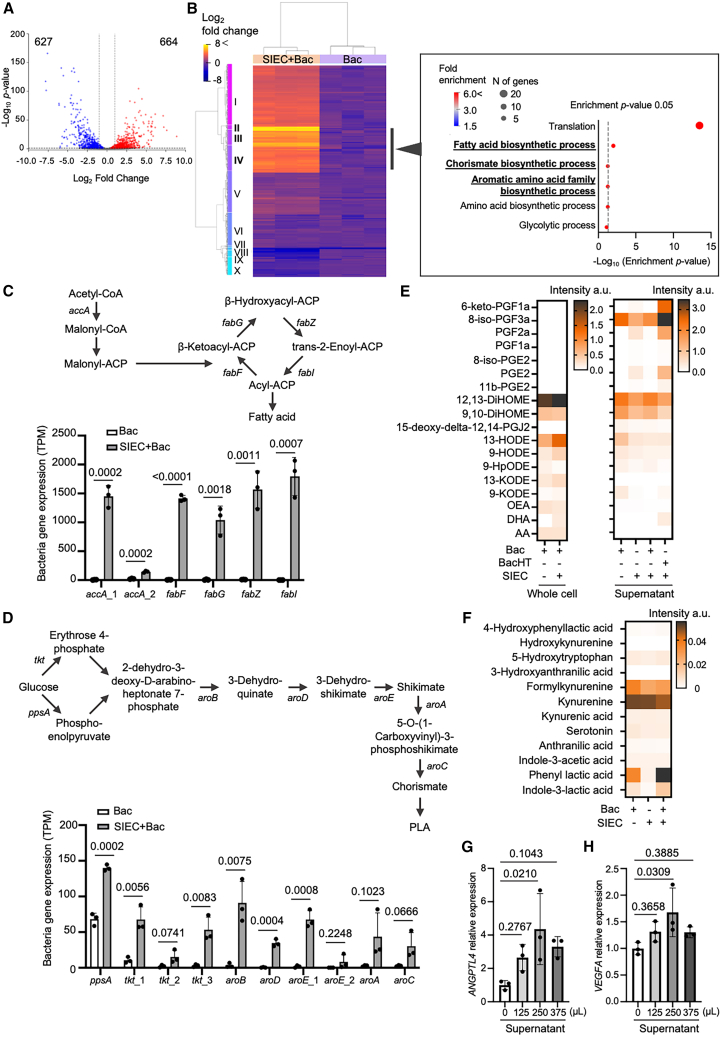
Aromatic amino acid and fatty acid biosynthesis modulation in *L. plantarum* via host epithelial interaction (A) Volcano plot illustrating DEGs in *L. plantarum* grown under monoculture and SIEC co-culture conditions; significantly regulated genes are indicated (FDR <0.05). (B) Heatmap shows bacterial DEGs involved in metabolic pathways under monoculture and SIEC co-culture conditions (adjusted *p* < 0.05; *n* = 3). Top enriched metabolic pathways indicated on the right (FDR <0.05). (C) Fatty acid biosynthesis pathway illustrates the significant upregulation of enzymes during co-culture, including acetyl-CoA carboxylase subunit A (*accA*), 3-oxoacyl-ACP synthase II (*fabF*), 3-oxoacyl-ACP reductase (*fabG*), (3R)- hydroxymyristoyl-ACP dehydratase (*fabZ*), and enoyl-ACP reductase (*fabI*). (D) Aromatic amino acid biosynthesis (shikimate) pathway highlighting the transcriptional induction of genes associated with phenyllactic acid (PLA) synthesis, including phosphoenolpyruvate synthase (*ppsA*), transketolase (*tkt*), 3-dehydroquinate synthase (*aroB*), 3-dehydroquinate dehydratase (*aroD*), shikimate dehydrogenase (*aroE*), 3-phosphoshikimate 1-carboxyvinyltransferase (*aroA*), and chorismate synthase (*aroC*). (E and F) Heatmaps show the relative abundances of lipid metabolites (E) and aromatic derivatives (F) detected in bacteria and supernatants. Data are presented as mean values from three independent biological replicates (*n* = 3) measured with LC-MS/MS. Statistical analysis was conducted using one-way ANOVA with Tukey’s test. (G and H) RT-qPCR analysis of *ANGPTL4* (G) and *VEGFA* (H) expression in SIECs treated with the exact volumes (125 μL, 250 μL, and 375 μL) of bacterial culture supernatants. Each data point represents the mean of technical triplicate RT-qPCR measurements performed using RNA extracted from cells pooled from two wells. Data are presented as mean ± SD from three independent biological replicates (*n* = 3), each measured in technical triplicate. Statistical significance was determined using one-way ANOVA followed by Dunnett’s multiple comparisons test.

We investigated the mechanisms by which *L. plantarum* modulates host metabolism by comparing bacterial transcriptomes between monoculture and co-culture with SIECs. In total, 1,291 DEGs were uniquely detected under co-culture conditions, including a cluster exhibiting significant upregulation (log_2_ fold change >5) compared to the levels under monoculture (Figures 4A, 4B, Tables S5, and S6). Principal component analysis revealed a clear separation between the two conditions, underscoring distinct transcriptomic profiles induced in *L. plantarum* (Figure S7A). Enrichment analysis revealed the upregulation of genes involved in fatty acid biosynthesis, notably including acetyl-CoA carboxylase subunit A (*accA*), 3-oxoacyl-ACP synthase II (*fabF*), 3-oxoacyl-ACP reductase (*fabG*), (3R)-hydroxymyristoyl-ACP dehydratase (*fabZ*), and enoyl-ACP reductase (*fabI*) (Figure 4C). These genes encode key enzymes responsible for the sequential conversion of acetyl-CoA to malonyl-CoA, β-ketoacyl-ACP, and ultimately, acyl-ACP, which are essential precursors for *de novo* fatty acid synthesis.^43^^,^^44^ In addition, genes associated with central carbon metabolism (phosphoenolpyruvate synthase [*ppsA*] and transketolase [*tkt*]) and the shikimate pathway (3-dehydroquinate synthase [*aroB*], 3-dehydroquinate dehydratase [*aroD*], shikimate dehydrogenase [*aroE*], 3-phosphoshikimate 1-carboxyvinyltransferase [*aroA*], and chorismate synthase [*aroC*]) were upregulated, along with genes involved in phenylalanine biosynthesis (Figure 4D).^45^ These transcriptomic profiles suggest that co-culture with SIECs triggers metabolic reprogramming in *L. plantarum*, promoting both lipid biosynthesis and aromatic amino acid metabolism, potentially enhancing the bacteria’s capacity to produce bioactive compounds relevant to host interactions.

We next profiled the bacterial metabolites using metabolomic and lipidomic analyses of both bacterial lysates and culture supernatants obtained from *L. plantarum* monocultures and co-cultures with SIECs (Figures 4E, 4F, S7B–S7F, S8A–S8E, Tables S7, and S8). Metabolomic analysis revealed substantial bacterial consumption of water-soluble compounds (Figures 4F, S7B–S7F, S8A–S8C, and Table S7), such as nucleic acids and sugars (Figures S7B and S7C), under bacterial monoculture and SIEC+Bac conditions, as evidenced by a marked depletion of these compounds compared to their levels under SIEC conditions. Most amino acids showed little difference among the conditions (Figure S8A). Of particular interest, aromatic amino acid derivatives such as phenyllactic acid (PLA) were elevated in SIEC+Bac relative to monocultured bacteria, with the difference being significant for PLA (*p* < 0.001, Figure S8B). This increase in PLA was likely linked to the upregulation of the shikimate pathway as revealed by RNA sequencing analysis (Figure 4D). SCFAs, including propionic acid and butyric acid, were notably reduced in SIEC and SIEC+Bac, but not in monocultured bacteria (Figure S7D), suggesting active cellular utilization. The reduction in butyrate under co-culture conditions likely resulted from enhanced uptake and metabolism by SIECs, as butyrate is a major energy source for intestinal epithelial cells.^46^ In contrast, acetic acid was accumulated in monocultured bacteria and reduced under SIEC+Bac conditions, suggesting altered fermentation dynamics driven by host interactions. As for other compounds, most water-soluble metabolites, such as vitamins and organic acids, showed little differences among the conditions (Figures S4F, S7E, S7F, S8C, and S8D).

Lipidomic profiling identified a total of 196 metabolites, among which six bioactive compounds (i.e., 12,13-dihydroxy-9Z-octadecenoic acid [12,13-diHOME], 9,10-dihydroxy-12Z-octadecenoic acid [9,10-diHOME], 13-hydroxyoctadeca-9Z,11E-dienoic acid [13-HODE], 9-hydroxyoctadeca-10E,12Z-dienoic acid [9-HODE], 9-hydroperoxyoctadeca-10E,12Z-dienoic acid [9-HpODE], and oleoylethanolamide [OEA]) were consistently detected in both bacterial lysates and supernatants exclusively under SIEC+Bac conditions (Figure S8E and Table S8). Notably, 12,13-diHOME, 9,10-diHOME, 13-HODE, and 9-HODE are recognized as potent PPARG agonists,^47^^,^^48^^,^^49^ suggesting that bacterial metabolic activity during co-culture may promote host nuclear receptor signaling. Collectively, these findings demonstrate that co-culture with SIECs reprograms *L. plantarum* metabolism, inducing PLA production and modestly increasing the levels of constitutively produced lipid mediators such as 12,13-diHOME and 9,10-diHOME. These compounds are poised to modulate host epithelial function via distinct signaling pathways such as PPARG.

Finally, to investigate how *L. plantarum* metabolites modulate host metabolic signaling, we examined their effects on PPARG signaling pathways in SIECs. SIECs were exposed to culture supernatants from live *L. plantarum* using increasing volumes of bacterial supernatant (125, 250, and 375 μL) (Figures 4G and 4H). The expression of angiopoietin-like 4 (*ANGPTL4*) and vascular endothelial growth factor A (*VEGFA)*, which are key downstream targets of PPARG signaling,^50^^,^^51^ was significantly upregulated in a dose-dependent manner (Figures 4G and 4H). In contrast, *ANGPTL4* and *VEGFA* expression was not upregulated in SIEC+BacHT (Figure S9A). These results indicate that bacterial metabolites preferentially activate the PPARG axis in SIECs. To validate this pathway specificity, SIECs were treated with individual lipid metabolites. Among the compounds tested, 12,13-diHOME and 9,10-diHOME showed a tendency to induce *ANGPTL4* and *VEGFA* expression, with 12,13-diHOME exhibiting a more pronounced, concentration-dependent trend (Figures S9B and S9C), supporting its potential role as a bacterial modulator of host PPARG signaling. To further assess the involvement of PPARG signaling, we performed pharmacological inhibition experiments using a PPARG antagonist. Under conditions in which SIECs were exposed to a variety of bacterial metabolic environments, *ANGPTL4* and *VEGFA* expression were significantly upregulated (Figure S9A). However, under the experimental conditions used for the inhibition assays, treatment with individual PPARG agonistic metabolites alone did not reproducibly induce *ANGPTL4* or *VEGFA* expression in SIECs, precluding a clear assessment of antagonist-mediated suppression (Figures S9D and S9E). This variability suggests that the activation of PPARG-related transcriptional responses in SIECs depends on the broader metabolic context, such as the combinatorial bacterial metabolic environment generated by live bacteria, rather than on a single metabolite acting in isolation.

Together, these results suggest that metabolites produced by live *L. plantarum* selectively stimulate PPARG-mediated transcriptional responses in epithelial cells. Given the role of PPARG in regulating lipid metabolism, oxidative stress responses, and epithelial barrier integrity, particularly under hypoxic conditions,^52^ its activation by bacterial metabolites may represent a key mechanism through which probiotic or postbiotic strains promote epithelial homeostasis and metabolic resilience.

## Discussion

This study uncovered fundamental differences in host responses to *L. plantarum* JCM 1149ᵀ, depending on bacterial viability. Using a controlled microfluidic co-culture system, we demonstrated that live bacteria orchestrate metabolic reprogramming in epithelial cells, characterized by a hypoxia-associated metabolic shift independent of HIF1A upregulation, along with the activation of the PPARG axis. In contrast, heat-treated bacteria preferentially engage innate immune pathways through NFKB signaling. These divergent outcomes demonstrate that the physiological impact of a bacterial strain depends not only on its genetic background but also critically on its viability—namely, whether the cells are metabolically active or inactivated by processing. This distinction underscores the importance of assessing viability-dependent functions when evaluating microbial effects on host metabolism and immunity, and when considering the appropriate application of probiotics and postbiotics for modulating host physiology and maintaining gut homeostasis.

Co-culture with live *L. plantarum* reduced oxygen tension in the epithelial microenvironment, suggesting a glycolytic shift in SIECs. This metabolic adaptation was characterized by the upregulation of key glycolytic enzyme genes and the downregulation of mitochondrial metabolic genes. Notably, this response occurred independently of HIF1A induction, as neither HIF1A transcript nor protein levels were altered, but was accompanied by the increased expression of *MXI1*, a Myc-associated repressor, suggesting a c-Myc-dependent, HIF-independent hypoxic adaptation.^53^ This glycolytic shift, characterized by enhanced glycolysis and suppressed mitochondrial gene expression, is consistent with the metabolic reprogramming typically observed under hypoxic stress.^54^ Although our data indicate that the observed metabolic reprogramming occurs independently of canonical HIF1A induction, this does not exclude a potential role for HIF1A at the level of protein stabilization or subcellular localization. Indeed, further investigation of HIF1A localization is warranted to distinguish between classical and non-canonical hypoxic signaling mechanisms. Notably, in our anaerobic chamber experiments, the expression of LDHA, a canonical HIF1A target, was not increased despite enhanced lactate production. This result suggests that artificial hypoxia induced by uniform oxygen deprivation does not fully recapitulate the signaling context generated by localized, bacteria-driven oxygen depletion. We speculate that subtle and spatially restricted hypoxia created by bacterial metabolic activity may differentially regulate hypoxic signaling pathways compared with experimentally imposed hypoxia. To dissect the mechanistic differences between these natural and artificial hypoxic conditions, future studies should conduct nuclear-cytoplasmic fractionation analyses to directly assess HIF1A localization and activation status. In line with these metabolic adaptations, TEM observations showed preserved membrane integrity of intracellular organelles, suggesting a potential shift toward lipid biosynthesis under such conditions. Together, these findings underscore the critical role of bacterial metabolic activity in modulating host cellular energy pathways.

Transcriptomic analysis of *L. plantarum* during co-culture revealed robust upregulation of genes involved in *de novo* fatty acid biosynthesis and the shikimate pathway, including those required for the synthesis of aromatic amino acid derivatives such as PLA. These metabolic shifts in *L. plantarum* are likely shaped by host-derived cues. Specifically, hypoxia-associated glycolytic shift in SIECs increases the availability of extracellular metabolites, including SCFAs, which may serve as both carbon sources and signaling molecules for the bacteria. In particular, SCFAs are known to stimulate bacterial fatty acid biosynthesis under nutrient-limited conditions,^38^^,^^50^ providing a plausible mechanism for the observed upregulation of bacterial fatty acid biosynthesis genes (*accA, fabG, fabZ, fabF*, and *fabI*). Although we did not directly quantify intracellular NADH levels in *L. plantarum*, a previous study indicated that bacterial glycolysis and the assimilation of extracellular metabolites such as lactate can elevate NADH pools via NAD^+^-dependent lactate dehydrogenases.^31^ This redox shift may in turn promote the reductive transformation of aromatic pyruvates, such as indolepyruvate and phenylpyruvate, into their corresponding aryllactates (indole-3-lactic acid and PLA) through NADH-dependent dehydrogenases. Given the glycolytic reprogramming observed in SIECs and the potential accumulation of lactate, host-derived metabolites possibly contribute to shaping the metabolic profile of *L. plantarum*. Taken together, these observations suggest that host-associated hypoxic and glycolytic conditions may modulate *L. plantarum* metabolism in a manner that promotes the synthesis of metabolites involved in immune regulation and epithelial barrier function. Although the present study primarily focused on the effects of *L. plantarum* on the host, including the induction of hypoxia and the production of PPARG-agonistic metabolites, reciprocal influences of the host on bacterial metabolism should also be considered. Culturing *L.* *plantarum* in conditioned media derived from hypoxic SIECs will be necessary in future studies to elucidate how host-driven environmental cues modulate bacterial metabolic programs.

In this study, 12,13-diHOME and 9,10-diHOME were examined as candidate lipid mediators based on their detection in the culture supernatant under co-culture conditions, suggesting a potential association with PPARG signaling. Notably, 12,13-diHOME has been implicated in immune regulation, including the modulation of inflammatory responses and allergic sensitization.^55^ When applied individually to SIECs, these compounds induced *ANGPTL4* and *VEGFA* expression; however, the magnitude and reproducibility of these responses varied across experiments. This variability suggests that stimulation with a single lipid mediator is insufficient to consistently recapitulate the transcriptional responses observed under SIEC+Bac conditions. In contrast, under co-culture with live bacteria—where epithelial cells were exposed to a complex mixture of bacterial metabolites—PPARG downstream gene expression was robustly induced. Together, these findings suggest that PPARG activation in epithelial cells arises from a combinatorial metabolic environment in which multiple bacterial-derived signals act in concert. Moreover, this lipid-centric mechanism may not be exclusive. Voltan et al. (2008) showed that *Lactobacillus crispatus*-derived hydrogen peroxide can serve as a redox signal to activate PPARG in intestinal epithelial cells.^56^ While hydrogen peroxide production was not assessed in our system, it is plausible that oxidative metabolites generated by live *L. plantarum* contribute to or enhance PPARG activation, potentially acting in concert with lipid mediators. These findings collectively underscore the multifaceted ways in which metabolites from viable bacteria modulate host nuclear receptors such as PPARG, reinforcing the importance of microbial metabolic activity in host-microbe interactions. In addition, PPARG can interact with NFKB to suppress inflammation by promoting the nuclear export of NFKB subunit p65, inhibiting NFKB transcriptional activity, and inducing proteasome-dependent degradation.^57^^,^^58^ Considering that NFKB activation observed during co-culture with heat-killed bacteria was not detected in co-culture with live bacteria, we suggest that PPARG-mediated anti-inflammatory effects explain this difference.

In contrast, the NFKB activation observed in response to heat-treated *L. plantarum* is primarily mediated through TLR4 signaling. While heat treatment led to the release of nucleic acids, these intracellular components did not elicit *TNFA* or *CCL4* expression, suggesting that the immunostimulatory effects are rather driven by structural changes on the bacterial cell surface. This is consistent with the altered composition and exposure of cell wall components following heat treatment. Bacterial surface structures, including lipoteichoic acids and extracellular polysaccharides, can act as pathogen-associated molecular patterns primarily recognized by TLR2 on host cells.^17^^,^^59^ Specifically, *L. plantarum* JCM 1149^T^, the strain used in our study, reportedly produces large amounts of extracellular polysaccharides, which can induce pro-inflammatory cytokines such as interleukin-6, -10, and −12.^60^ Additionally, lipoteichoic acids from *L. plantarum* JCM 1149^T^ exhibit strong IgA-inducing activity in mucosal tissues,^61^ highlighting their immunogenic potential. These findings suggest that the TLR-mediated recognition of surface components is an important mechanism underlying the immune response to *L. plantarum*. However, siRNA-mediated knockdown and neutralizing antibody treatment revealed that TLR4 also contributed to *TNFA* or *CCL4* transcriptional expression in heat-treated cells, suggesting that additional ligands are involved. Although we were unable to reliably detect TNFA and CCL4 at the protein level because of their low abundance in the culture supernatant, the consistent transcriptional induction supports a role for TLR4-dependent signaling under these conditions. Among known TLR4 ligands, lipopolysaccharide^62^^,^^63^ is characteristic of gram-negative bacteria and is therefore unlikely to be relevant in the current context. In contrast, extracellular heat shock proteins,^64^^,^^65^ which are released or exposed upon heat treatment and are also known TLR4 agonists, represent plausible candidates. A previous study has shown that in *B. longum* BB536, certain heat shock proteins, such as IbpA, retain their cytokine production capacity even after heat treatment at 70 °C but lose this activity at 90 °C.^66^ Notably, the heat treatment in our study was conducted at 70 °C, supporting the possibility that heat shock proteins contribute to the observed TLR4-mediated immune activation. Moreover, previous studies have reported that *L. fermentum* CECT5716 induces TLR9 signaling and increases the phosphorylation of the NFKB inhibitor IKBA.^67^ Notably, viable *L. fermentum* CECT5716 induced significantly higher TLR9 activation than heat-inactivated bacteria, suggesting that bacterial viability can critically influence TLR pathway engagement and downstream NFKB regulation. TLR9 is known to recognize unmethylated CpG motifs in bacterial DNA within endosomal compartments. However, in our experimental system, the stimulation of SIECs with bacterial-derived nucleic acid mixture did not alter the expression of NFKB downstream genes, such as *TNFA* or *CCL4*. These observations suggest that, although TLR9-dependent NFKB modulation has been described for other bacterial species, the contribution of TLR9 signaling is likely minimal in our model. Instead, our findings support the notion that NFKB activation in response to heat-treated *L. plantarum* is primarily driven by viability-dependent alterations in bacterial surface structures, which may expose or render accessible bacterial components capable of engaging TLR4 signaling. Further investigation is needed to identify the precise ligands involved in this atypical TLR4 signaling.

In conclusion, our findings highlight a paradigm shift in understanding how bacterial viability governs host epithelial responses. We demonstrate that live *L. plantarum* induces localized hypoxia and metabolic reprogramming in epithelial cells, leading to a glycolytic shift and PPARG activation associated with enhanced epithelial barrier-supportive programs. This response arises from a complex metabolic environment generated by viable bacteria rather than from a single defined metabolite acting in isolation. In contrast, heat-treated bacteria preferentially elicit immunostimulatory responses through TLR4-dependent NFKB activation, likely driven by viability-dependent alterations in bacterial surface structures and stress-associated components. These findings underscore that bacterial viability fundamentally shapes the nature of host signaling pathways engaged, resulting in distinct metabolic versus inflammatory outcomes. Importantly, this viability-dependent bifurcation provides a conceptual framework for the rational application of probiotics and postbiotics. While viable probiotics may be leveraged to modulate epithelial metabolism and barrier function through metabolic and hypoxia-associated pathways, non-viable bacterial preparations may offer complementary opportunities for immune modulation. Together, our study emphasizes the need to consider bacterial viability as a critical determinant of host-microbe interactions and highlights the potential for tailoring microbial-based interventions to achieve specific epithelial metabolic or immunological outcomes.

### Limitations of the study

While our microfluidic co-culture system effectively recapitulates direct interactions between live *L. plantarum* and epithelial cells, including localized oxygen depletion and hypoxia-associated metabolic reprogramming, several limitations should be considered when interpreting our findings.

First, our data indicate a close interplay between hypoxia-induced metabolic reprogramming in epithelial cells and bacterial metabolic activity. However, due to the reciprocal nature of host-microbe interactions within the co-culture system, it remains challenging to definitively resolve the causal directionality between epithelial metabolic changes and bacterial metabolic responses. Dissecting whether epithelial hypoxia-driven metabolic reprogramming shapes bacterial metabolism or whether bacterial metabolic activity initiates epithelial reprogramming will require experimental strategies that enable temporal or metabolic uncoupling of host and bacterial responses. Second, the present study focused on epithelial cell-intrinsic responses to bacterial viability. Interactions between epithelial and immune cells, which play central roles in shaping mucosal immune responses, were not addressed in this model. Incorporating immune cell populations will be important to determine how epithelial metabolic and inflammatory programs influenced by bacterial viability are integrated into tissue-level immune regulation. Third, the present study was conducted using a single bacterial strain (*L. plantarum* JCM 1149ᵀ) and a single epithelial cell model (SIECs). Although SIECs provide a robust and reproducible platform for co-culture experiments, the observed responses reflect outcomes specific to this host-microbe pairing. In addition, this study used only male pig-derived cells, and sex-dependent differences may limit the generalizability of the findings. Given the strain-dependent nature of probiotic effects and potential interspecies differences in epithelial signaling, future studies should employ multiple bacterial strains and epithelial models derived from diverse host species, including humans, to assess the generalizability of our findings. Despite these limitations, our study provides a representative framework illustrating how bacterial viability differentially shapes epithelial metabolic and immune responses. These insights may inform the rational design of microbiota-based interventions, guiding the use of viable probiotics for epithelial metabolic support and non-viable bacterial preparations for targeted immune modulation.

## Resource availability

### Lead contact

Requests for further information and resources should be directed to and will be fulfilled by the lead contact, Keita Nishiyama (keita.nishiyama.a6@tohoku.ac.jp).

### Materials availability

This study did not generate new unique reagents.

### Data and code availability

• RNA-seq data from SIECs and *L. plantarum* have been deposited in the DDBJ/NCBI BioProject database under accession numbers PRJDB35408 and PRJDB35409. Metabolomic data generated from LC-MS/MS analyses are provided in the Tables S7 and S8.
• The code used for transcriptome analysis is available at GitHub (https://github.com/yutatakada38-cyber/Matsumoto-et-al.-2026_RNA-seq-analysis).
• Any additional information required to reanalyze the data reported in this paper is available from the lead contact upon request.

## Acknowledgments

We gratefully acknowledge Dr. Ayako Miyazaki and Dr. Kotaro Miyazawa (National Institute of Animal Health, 10.13039/501100007172NARO) for kindly providing the swine intestinal epithelial cells (SIECs) used in this work. We also acknowledge Ms. Yui Asano (Laboratory of Animal Food Function, Graduate School of Agricultural Science, 10.13039/501100006004Tohoku University) for kindly conducting the formal analysis. This study was supported by a Grant-in-Aid for Scientific Research (B) (23K27051, K.N.) and a Grant-in-Aid for Scientific Research (A) (23H00354, H.K.) from the 10.13039/501100001691Japan Society for the Promotion of Science (10.13039/501100001691JSPS), a Development of Innovative Technology grant (JPJ007097, H.K.) from the 10.13039/501100007173Bio-oriented Technology Research Advancement Institution (10.13039/501100007173BRAIN), as well as by the 10.13039/100015103Japan Racing Association (H.K.), the 10.13039/100016974Food Science Institute Foundation (Ryosyoku-kenkyukai, H.K.), the Association for Research on Lactic Acid Bacteria (H.K.), 10.13039/100009619Japan Agency for Medical Research and Development (10.13039/100009619AMED) grant (JP21zf0127001, H.K.), and the 10.13039/100008732Uehara Memorial Foundation (K.N.).

## Author contributions

Conceptualization: K.M., Y.Tk., Y.Ts., T.H., K.N., and H.K.; methodology and investigation: K.M., Y.Tk., K.S., Y.I., H.Y., M.T., N.M., T.S., and F.N.; formal analysis: K.M., Y.Tk., K.S., and Y.I.; resources: J.Y., L.A., W.I.-O., J.V., F.N., Y.Ts., T.H., K.N., and H.K.; visualization: K.M., Y.Tk., and K.N.; writing – original draft: K.M., Y.Tk., K.S., N.M., and K.N.; writing – review and editing: J.Y., L.A., W.I.-O., M.H., J.V., F.N., Y.Ts., T.H., K.N., and H.K.; funding acquisition: K.N. and H.K.; supervision: K.N. and H.K.; project administration: T.H., K.N., and H.K.

## Declaration of interests

Kazuhiro Sonomura, Mikako Takahashi, and Toyoyuki Hashimoto are employees of Shimadzu Corp. Junya Yamamoto and Yuji Tsujikawa are employees of ITOEN, Ltd. The remaining authors declare no competing interests.

## Declaration of generative AI and AI-assisted technologies in the writing process

During the preparation of this work, the authors used ChatGPT (OpenAI, version 5.2) in order to improve the readability and language of the manuscript. After using this tool or service, the authors reviewed and edited the content as needed and take full responsibility for the content of the publication.

## STAR★Methods

### Key resources table

**Table:** 

REAGENT or RESOURCE	SOURCE	IDENTIFIER
**Antibodies**		

HIF-1 alpha Rabbit PolyAb	Proteintech	Cat #: 20960-1-AP
LDHA Polyclonal Antibody	Thermo Fisher Scientific	Cat #: PA5-23036
Beta Actin Rabbit PolyAb	Proteintech	Cat #: 20536-1-AP
HRP-conjugated Goat Anti-Rabbit IgG (H + L)	Proteintech	Cat #: SA00001-2
Mouse IgG2b isotype control Monoclonal antibody	Proteintech	Cat #: 66360-3-IG
TLR4 Monoclonal antibody	Proteintech	Cat #: 66350-1-IG

**Bacterial and virus strains**		

*Lactiplantibacillus plantarum*	Japan Collection of Microorganisms (RIKEN BRC)	JCM1149^T^

**Chemicals, peptides, and recombinant proteins**		

TRI Reagent	Molecular Research Center	Cat #: TR118
Chloroform	FUJIFILM Wako Pure Chemical	Cat #: 038-02606
GW9662	FUJIFILM Wako Pure Chemical	Cat #: 075-05611
Rosiglitazone	FUJIFILM Wako Pure Chemical	Cat #: 184-02651
(±)9-HODE	Cayman Chemical	Cat #: 38400
(±)13-HODE	Cayman Chemical	Cat #: 38600
(±)9, 10-DiHOME	Cayman Chemical	Cat #: 53400
(±)12, 13-DiHOME	Cayman Chemical	Cat #: 10009832

**Critical commercial assays**		

NEBNext Poly(A) mRNA Magnetic Isolation Module (NEB #E7490)	New England Biolabs	Cat #: E7490
NEBNext Ultra RNA Library Prep Kit for Illumina (NEB #E7530)	New England Biolabs	Cat #: E7530
Ribo-Zero Plus rRNA Depletion Kit	Illumina	Cat #: 20040526
NEBNext Ultra II Directional RNA Library Prep Kit	New England Biolabs	Cat #: E7760S
RNeasy Mini Kit (50)	QIAGEN	Cat #: 74104
Trans-Blot Turbo RTA Transfer Kit, LF PVDF	Bio-Rad	Cat #: 1704274
ISOSPIN Fecal DNA	NIPPON GENE	Cat #: 315-08621
PrimeScript^TM^ RT reagent Kit with gDNA Eraser (Perfect Real Time)	Takara Bio	Cat #: RR047A

**Deposited data**		

Raw data	This paper	PRJDB35408, PRJDB35409
Code used for transcriptome analysis	This paper	https://github.com/yutatakada38-cyber/Matsumoto-et-al.-2026_RNA-seq-analysis

**Experimental models: Cell lines**		

Swine intestinal epithelial cells (SIECs), derived from small intestinal crypts of a two-week-old male piglet	Originally described in Matsumoto et al., 2024^43^	https://doi.org/10.12938/bmfh.2024-0046

**Oligonucleotides**		

siRNA targeting sequences, see Table S9	This study	N/A
Primers, see Table S10	see Table S10	see Table S10

**Software and algorithms**		

GraphPad Prism (version 10)	N/A	N/A
FastQC (version 0.11.7)	N/A	https://www.bioinformatics.babraham.ac.uk/projects/fastqc/
Trimmomatic (version 0.38)	N/A	N/A
HISAT2 (version 2.1.0)	N/A	N/A
featureCounts (version 1.6.3)	N/A	N/A
DESeq2 (R v4.2.3, v1.36.0)	N/A	N/A
scikit-learn (Python v3.10, v1.0.2)	N/A	N/A
Kyoto Encyclopedia of Genes and Genomes (KEGG) pathways	N/A	https://www.genome.jp/kegg/kegg_ja.html
DAVID	N/A	https://davidbioinformatics.nih.gov
ImageJ	N/A	https://imagej.net/ij/

**Other**		

Metabolomic data generated from LC-MS/MS analyses	see Tables S7 and S8	see Tables S7 and S8

### Experimental model and study participant details

#### *L. plantarum* JCM 1149^T^ culture

*L. plantarum* JCM 1149^T^ was obtained from the RIKEN BioResource Research Center (JCM, Tokyo, Japan) and routinely cultured anaerobically at 37 °C in de Man, Rogosa, Sharpe (MRS) broth or on MRS agar plates (Difco, Detroit, MI, USA).

#### SIEC culture

SIECs were previously derived from the intestinal crypts of a two-week-old male Duroc piglet.^43^ Cells were originally provided by the National Agriculture and Food Research Organization, National Institute of Animal Health (NARO-NIAH, Japan). Cells were routinely maintained in growth medium consisting of Dulbecco’s modified Eagle’s medium (DMEM)/F12 supplemented with 1× insulin–transferrin–selenium (Merck/Millipore Sigma, Burlington, MA, USA), 5 ng/mL recombinant human epidermal growth factor (Merck/Millipore Sigma), 10% heat-inactivated fetal bovine serum, and 1% penicillin–streptomycin (Thermo Fisher Scientific, Waltham, MA, USA).

### Method details

#### *L. plantarum* JCM 1149^T^ heat inactivation

To prepare heat-treated bacteria, the strain was subcultured three times consecutively in MRS broth under anaerobic conditions at 37 °C for 16 h per passage. Then, bacterial cells were harvested by centrifugation, washed three times with sterile phosphate-buffered saline (PBS), and suspended in PBS. The cells were heat-treated by incubation in a water bath at 70 °C for 90 min and then diluted with 0.4× mGAM prepared by diluting mGAM to 40% with Disintegration Test Solution No. 2 (0.2 mol/L KH_2_PO_4_ and 0.2 mol/L NaOH) to a final concentration of 2.5 × 10⁹ cells/mL. Aliquots of the heat-treated bacterial preparation were stored at −20 °C until use in co-culture experiments.

#### Co-culture of SIECs with live or heat-treated *L. plantarum* JCM 1149^T^

SIECs were seeded at a density of 6.8 × 10⁵ cells/mL into 12-well Transwell cell culture inserts (Greiner Bio-One, Tokyo, Japan) pre-coated with 250 μL of a collagen solution (3 mg/mL type I powdered collagen derived from porcine dermis; Nippi, Tokyo, Japan), prepared by diluting the collagen in sterile 5 mM acetic acid at a ratio of 1:300. After 30 min of incubation, the inserts were washed twice with PBS.

For co-culture with live *L. plantarum* JCM 1149^T^, we employed a custom co-culture device (Shimadzu, Kyoto, Japan) capable of maintaining anaerobic conditions on the apical side and aerobic conditions on the basal side by hermetically sealing the Transwell insert with a culture cup. Two days after SIEC seeding, the inserts were transferred into the device. The basal chamber was filled with DMEM (low glucose, with pyruvate; Thermo Fisher Scientific) supplemented with 10% fetal calf serum (FCS). On the apical side, the culture medium was replaced with 0.4× mGAM medium and incubated at 37 °C overnight to acclimate the cells. Live L. *plantarum* was suspended in 0.4× mGAM and adjusted to 2.8 × 10^5^ CFU/mL (OD_600_ = 0.001) or 4.0 × 10⁶ (CFU)/mL (OD_600_ = 0.01), and 500 μL of the suspension was added to the apical compartment. Control wells were filled with an equal volume of 0.4× mGAM alone. During co-culture, fresh 0.4× mGAM was continuously supplied to the apical side at a flow rate of 5 μL/min. TEER was monitored throughout the experiment to assess epithelial barrier integrity. After 48 h of incubation at 37 °C, both apical and basal media were collected for CFU quantification on MRS agar plates. Cells remaining on the Transwell inserts were lysed with 500 μL of TRI Reagent (Molecular Research Center, Cincinnati, OH, USA). The media were centrifuged at 15,000 rpm, 4 °C for 10 min to separate bacterial pellets and supernatants, which were stored at −80 °C until use.

For co-culture with heat-treated *L. plantarum*, SIECs were seeded and maintained under the above conditions. Two days after seeding, the basal side was filled with low-glucose DMEM supplemented with 10% FCS, the apical medium was replaced with 0.4× mGAM, and the plates were incubated overnight at 37 °C. Heat-treated *L. plantarum* was adjusted to an OD_600_ of 0.01 and applied to the apical side. Control wells were filled with an equal volume of 0.4× mGAM medium. After 48 h of static incubation at 37 °C, apical and basal media were collected, and all samples were stored at −80 °C until analysis.

#### Anaerobic culture of SIECs using an anaerobic chamber

SIECs were seeded at a density of 6.8 × 10⁵ cells/mL into 12-well Transwell cell culture inserts (Greiner Bio-One) and cultured under standard conditions. Transwell plates were transferred into the anaerobic chamber (Coy Laboratory Products, MI, USA), and cells were maintained at 37°C using an in-chamber incubator (MITSUBISHI ELECTRIC ENGINEERING, Tokyo, Japan). Cells were then cultured for 48 h under anaerobic conditions. The chamber atmosphere was maintained at <0.01% O_2_ with a gas mixture of N_2_ 80%/H_2_ 5%/CO_2_ 15%.

#### RNA extraction

RNAs of SIECs and *L. plantarum* were extracted separately. To extract RNA from SIECs, the apical medium was removed, and the inserts were washed twice with PBS. Cells attached to the inserts were then harvested using TRIzol (Molecular Research Center, OH, USA). Each sample was added with 100 μL of chloroform (FUJIFILM Wako Pure Chemical, Osaka, Japan), vigorously mixed, and then incubated at room temperature for 3 min. The samples were then centrifuged at 15,000 rpm, 4 °C for 15 min. The aqueous phase of the supernatant was carefully transferred to a 1.5 mL microcentrifuge tube containing 3 μL of glycogen solution (20 mg/mL; Nacalai Tesque, Kyoto, Japan) and 200 μL of 2-propanol (FUJIFILM Wako Pure Chemical). After ethanol precipitation, the pellet was washed with 500 μL of 75% ethanol and centrifuged at 15,000 rpm, 4 °C for 20 min. The supernatant was discarded, and the pellet was air-dried at room temperature for 10 min before being resuspended in diethylpyrocarbonate-treated water. RNA concentration and purity were assessed using a NanoDrop One spectrophotometer (Thermo Fisher Scientific).

To extract RNA from *L. plantarum*, bacterial cells were harvested after both monoculture and co-culture. In the monoculture condition, bacteria were pre-cultured and subsequently incubated in 0.4× mGAM medium for 48 h. In the co-culture condition, bacterial pellets were obtained by centrifuging apical medium collected from co-cultures with SIECs. The pellets were treated with RNA Protect Bacteria Reagent (Qiagen, Hilden, Germany) and stored at −80 °C until RNA extraction. To digest the bacterial cell walls, the pellets were resuspended in TE buffer containing 15 mg/mL lysozyme (Sigma-Aldrich, St. Louis, MO, USA) and 1,000 U/mL mutanolysin (Sigma-Aldrich), and incubated at room temperature (20–25°C) for 1 h. Subsequently, 1.5 mg/mL Proteinase K (Qiagen) was added, followed by incubation for an additional 30 min. Total RNA was extracted using the RNeasy Mini Kit (Qiagen), in combination with the RNase-Free DNase Set (Qiagen), according to the manufacturer’s instructions.

#### Dual RNA sequencing and data analysis

RNA quality was assessed using a BioAnalyzer (Agilent Technologies, Santa Clara, CA, USA). For host epithelial cells, RNA libraries were prepared using the NEBNext Poly(A) mRNA Magnetic Isolation Module (NEB #E7490) and the NEBNext Ultra RNA Library Prep Kit for Illumina (NEB #E7530) (New England Biolabs, Ipswich, MA, USA). For bacterial RNA, the Ribo-Zero Plus rRNA Depletion Kit (Illumina, San Diego, CA, USA) and NEBNext Ultra II Directional RNA Library Prep Kit (New England Biolabs) were used. Sequencing was performed on an Illumina NovaSeq 6000 platform (Illumina), generating paired-end 150-bp reads.

For the epithelial cell libraries, approximately 4 Gb of sequencing data and an approximately 26.7 million reads per sample were obtained. For the bacterial libraries, approximately 1 Gb of sequencing data and an average of 6.7 million reads per sample were obtained. Sequencing quality was assessed using FastQC (v0.11.7), and adapters were trimmed using Trimmomatic (v0.38) with the following parameters: ILLUMINACLIP:2:30:10, LEADING:20, TRAILING:20, SLIDINGWINDOW:4:15, MINLEN:36, based on Illumina adapter sequences (AGATCGGAAGAGCACACGTCTGAACTCCAGTCA, AGATCGGAAGAGCGTCGTGTAGGGAAAGAGTGT). Reads were aligned to the reference genomes, Sscrofa11.1 (GCA_000003025.6) for swine cells and GCF_000143745.1 for *L. plantarum* JCM 1149^T^, using HISAT2 (v2.1.0). Read counts were generated using featureCounts (v1.6.3).Principal component analysis and differential expression analysis were performed using DESeq2 (R v4.2.3, v1.36.0) and scikit-learn (Python v3.10, v1.0.2). DEGs were identified by comparing each treatment condition (co-culture or heat-treated co-culture) with monoculture samples using a false discovery rate (FDR)-adjusted *p*-value threshold of <0.05 after Benjamini–Hochberg correction. Read counts were normalized to transcripts per million (TPM) by adding one to all values. For SIEC transcriptome analyses (Figures 2A and 3A), log_2_ fold changes were calculated relative to the mean of all samples. For metabolic and immune-related expression patterns (Figures 2B, 3B, S4B, and S6B) and for *L. plantarum* transcriptomes, log_2_ fold changes were calculated relative to the monoculture means. Group-specific expression patterns were classified through hierarchical clustering using the Ward method, followed by enrichment analysis using Kyoto Encyclopedia of Genes and Genomes (KEGG) pathways (v112.0) for SIECs and Gene Ontology biological process terms for *L. plantarum*. Enrichment analyses were performed using DAVID (v2024q4). For L. *plantarum*, annotation was based on the file GCF_000143745.1_ASM14374v1_genomic.processed.gtf, and only genes registered in DAVID were used. In the analysis of metabolic and immune-related expression patterns, only DEGs and group means are presented. Gene sets for each pathway, including HIF-1 signaling, NFKB signaling, glycolysis/gluconeogenesis, the tricarboxylic acid cycle, oxidative phosphorylation, and TNF signaling pathways, were obtained from KEGG annotations for *Sus scrofa*. Mapping results, gene lists, and enrichment analyses are summarized in Tables S2, S4, S5, and S6. The code used for transcriptome analysis is available at GitHub (https://github.com/yutatakada38-cyber/Matsumoto-et-al.-2026_RNA-seq-analysis).

#### Nucleic acid extraction

Nucleic acids were extracted from live *L. plantarum* JCM 1149^T^. In brief, bacterial cultures were centrifuged to remove the supernatant. Nucleic acids were extracted from the pellets using ISOSPIN Fecal DNA (NIPPON GENE, Tokyo, Japan) according to the manufacturer’s instructions.

#### siRNA transfection

SIECs were seeded into 12-well cell culture plates (AGC TECHNO GLASS, Shizuoka, Japan) at a density of 3.0 × 10⁵ cells/mL and cultured under standard conditions. After 24 h, siRNAs targeting *TLR2* and *TLR4* were transfected into cells (Table S9). Cells were washed, and the culture medium was replaced with 900 μL per well of DMEM/F-12 supplemented with 10% FCS, 1% insulin–transferrin–selenium (×100; Thermo Fisher Scientific), and 5 ng/mL human epidermal growth factor (Sigma-Aldrich).

For transfection, 50 μL of Opti-MEM I Reduced-Serum Medium (Thermo Fisher Scientific) was mixed with 3.0 μL of Lipofectamine RNAiMAX Reagent (Thermo Fisher Scientific) and gently vortexed. In parallel, 50 μL of Opti-MEM I was mixed with 0.25 μL of each siRNA and then gently vortexed. The two solutions were mixed, gently vortexed again, and incubated at room temperature for 5 min for transfection complex formation. The complex (100 μL/well) was then added to the cells at a final siRNA concentration of 5 nM. As a negative control, Stealth RNAi Negative Control Duplexes (Thermo Fisher Scientific) were used at the same concentration. Transfected cells were incubated under standard culture conditions (37 °C, 5% CO_2_) for 4 days before harvesting.

#### Neutralizing antibody treatment

SIECs were seeded into 48-well cell culture plates (AGC TECHNO GLASS) at a density of 3.0 × 10^5^ cells/mL and cultured under standard conditions. SIECs were pretreated with an anti-TLR4 neutralizing antibody (Proteintech) or an isotype-matched control antibody (Proteintech) at a final concentration of 500 ng/mL. Antibodies were added 3 h prior to stimulation with the heat-treated bacterial suspension. Subsequently, cells were stimulated with BacHT suspension at the indicated dose for 48 h.

#### Quantitative reverse transcription PCR (RT-qPCR)

SIECs were seeded at a density of 6.8 × 10^5^ cells/mL into 12-well Transwell cell culture inserts (Greiner Bio-One) or 48-well cell culture plates (AGC TECHNO GLASS) and cultured under standard conditions. Then, the SIECs were treated with bacterial culture supernatant, bacterial culture suspension, purified bacterial nucleic acid mixture, or individual lipid compounds (rosiglitazone (FUJIFILM Wako Pure Chemical), 9-HODE, 13-HODE, 9,10-DiHOME, and 12,13-DiHOME (Cayman Chemical, Ann Arbor, MI, USA) for 48 h without co-culture device.

RNA was extracted using TRI Reagent (Molecular Research Center), and cDNA was synthesized using a PrimeScript RT Reagent Kit with gDNA Eraser (Perfect Real Time) (Takara Bio, Shiga, Japan), per the manufacturer’s instructions. qPCRs were run using TB Green Premix Ex Taq II (Takara Bio) in a CFX Connect Real-Time System (Bio- Rad Laboratories, Hercules, CA, USA). The primers used are listed in Table S10. Amplification was performed at 95 °C for 30 s and 40 cycles at 95 °C for 5 s and 60 °C for 30 s. Actin Beta (ACTB) was used as an internal control to normalize mRNA expression levels, as previously described.^43^

#### Western blot

Cells were lysed in RIPA lysis buffer supplemented with protease/phosphatase inhibitors (Atto, Tokyo, Japan). Lysates were clarified by centrifugation at 12000×g for 10 min at 4 °C. Protein samples were separated by SDS-PAGE on 10% polyacrylamide gels and transferred onto PVDF membranes (Bio-rad, CA, USA). The membranes were blocked with 5% skim milk in PBS for an hour or blocking buffer (Bio-rad) for 5 min at room temperature and then incubated with primary antibodies against ACTB (Proteintech, IL, USA), HIF1A (Proteintech), and LDHA (Thermo Fisher Scientific) at a dilution of 1:1000 overnight at 4 °C. ACTB was used as a loading control. After washing with TBS-T (Bio-rad), the membranes were incubated with HRP-conjugated secondary antibodies (Proteintech) at a dilution of 1:2000 for an hour at room temperature. Protein bands were visualized using Clarity Max Western ECL Substrate (Bio-rad) and detected with ChemiDoc Touch MP Imaging system (Bio-rad). Band intensities were quantified using ImageJ, and target protein levels were normalized to ACTB.

#### Electron microscopy

Cells were fixed in freshly prepared 2.5% glutaraldehyde diluted in PBS and incubated at 4 °C overnight. Then, the samples were washed with PBS, transferred into a 0.1 M cacodylate buffer (pH 7.4), and divided for SEM and TEM processing.

For SEM, samples were treated with 1% tannic acid to enhance conductivity, followed by post-fixation in 1% osmium tetroxide diluted in 0.1 M cacodylate buffer at 4 °C for 2 h. The samples were dehydrated through a graded ethanol series up to absolute ethanol and then infiltrated with *tert*-butyl alcohol. After freezing at −10 °C, the samples were dried using a vacuum freeze-dryer (VFD-21S; Vacuum Device, Ibaraki, Japan). The dried specimens were mounted on SEM stubs and coated with a thin layer of osmium using a conductive osmium coater (Neoc-ST; Meiwafosis, Tokyo, Japan). SEM images were acquired using an SU6600 scanning electron microscope (Hitachi High-Tech, Tokyo, Japan) at an acceleration voltage of 5 kV.

For TEM, samples were post-fixed in 1% osmium tetroxide diluted in 0.1 M cacodylate buffer at 4 °C for 2 h and then stained with 1% uranyl acetate for 20 min. The samples were dehydrated through a graded ethanol series up to absolute ethanol, followed by infiltration with QY-1 (n-butyl glycidyl ether; Nisshin EM, Tokyo, Japan) and increasing concentrations of epoxy resin mixed with QY-1, followed by 100% epoxy resin. The samples were embedded in pure epoxy resin and polymerized at 60 °C for 72 h. Ultrathin sections (∼80 nm) were prepared using an ultramicrotome (Leica UC7; Leica Microsystems, Wetzlar, Germany), mounted on copper grids (Veco, Eerbeek, the Netherlands), and stained with uranyl acetate and lead citrate for 10 min each. TEM images were acquired using a JEM-1400plus transmission electron microscope (JEOL, Tokyo, Japan) operated at 100 kV.

#### Metabolomic analysis

All data were acquired using on a liquid chromatography-mass spectrometry (LC-MS) system consisting of a Nexera UHPLC and LCMS-8050 (Shimadzu) with electrospray ionization under reaction monitoring mode. Metabolites in medium samples were analyzed using LC/MS/MS Method Package Cell Culture Profiling v2 and Short-Chain Fatty Acid and Lipid Mediator v3 (Shimadzu). The LCMS system was set up according to the optimized analytical conditions provided in each LC/MS/MS method package, including chromatographic separation, MS interface conditions, retention time, and ion transitions of each metabolite. Targeted metabolites are listed in Tables S7 and S8.

Medium samples (100 μL) were mixed with 300 μL of methanol, and 20 μL of internal standard solution (2 nM 2-isopropylmalic acid (Merck) in 0.01 mM HCl) was added. After centrifugation at 16,000 × *g*, 4 °C for 10 min, the supernatants were aliquoted and treated according to the respective LCMS analyses. For cell-culture profiling analysis, 40 μL of the supernatant was diluted with 10 μL of 0.01 mol/L HCl or 20 μL of the supernatant was diluted with 80 μL of 0.01 mM HCl, and 1 μL of the diluted samples was injected into the LC-MS system. For SCFA analysis, metabolites were derivatized with 3-nitrophenylhydrazine. The supernatant (50 μL) was mixed with reaction solution containing 50 μL of ultrapure water, 7.5% pyridine in methanol, 50 mM 3-nitrophenylhydrazine (Merck) in methanol, and 50 mM 1-ethyl-3-(3-dimethylamino-propyl)-carbodiimide (Merck) in methanol. The reaction mixture was incubated at 37 °C for 30 min. The reaction was stopped by adding 30 μL of 10% trifluoroacetic acid, and 3 μL of the mixture was injected into the LC-MS system. For lipid mediator analysis, another internal standard solution (6-keto-PGF1a-d4, PGF2a-d4, 15-HETE-d8: 60 ng/mL, OEA-d4: 12 ng/mL in methanol) was prepared. The supernatant (120 μL) was mixed with 10 μL of the internal standard and purified using solid phase extraction according to the manufacturer’s protocol. The purified samples were reconstituted in 20 μL of methanol, and 5 μL was injected into the LC-MS system. Metabolite levels were assessed by comparing the peak area ratio calculated by dividing the peak area of each metabolite with that of the internal standard.

#### Oxygen concentration measurement

SIECs were seeded into 12-well Transwell cell culture inserts at a density of 6.8 × 10^5^ cells/mL and cultured under standard conditions. To assess oxygen levels at the apical side of the epithelium, an optical oxygen sensor (OXY-1 ST trace; PreSens, Regensburg, Germany) was inserted into the adapter hole of the co-culture device under continuous medium flow. The sensor tip was carefully positioned in close proximity to the cell surface without disturbing the monolayer. Oxygen concentrations were continuously monitored over 48 h at 37 °C, enabling real-time and non-invasive monitoring of oxygen dynamics during epithelial cell culture and bacterial co-culture. Data were recorded and analyzed using the accompanying software, and oxygen levels were expressed in ppm (corresponding to dissolved oxygen concentrations of 0–45 mg/L or 0–1400 μmol/L). According to the manufacturer’s specifications, the sensor exhibits a fluctuation range of ±0.05% O_2_ at high oxygen concentrations or a relative error of <3% at low oxygen concentrations.

### Quantification and statistical analysis

Statistical analyses were performed using GraphPad Prism (version 10, GraphPad Software). Specific statistical tests are described in the figure legends. Statistical significance was set at *p* < 0.05.

## Supplemental information

Document S1. Figures S1–S9

Table S1. pH values of culture media under each condition, related to Figure 1B

Table S2. Gene list of cluster X enriched in glycolysis and HIF-1 signaling, related to Figure 2A

Table S3. Oxygen concentration measurements across culture conditions, related to Figure 2G, S4I

Table S4. Gene clusters (I-III) upregulated by heat-treated *L. plantarum*, related to Figure 3A

Table S5. DEGs in *L. plantarum* during co-culture with SIECs, related to Figure 4B

Table S6. Functional annotation of bacterial gene clusters, related to Figure 4B

Table S7. Quantification of water-soluble metabolites in culture supernatants, related to Figure 4F, S7B–F, S8A, S8C, S8D

Table S8. Quantification of lipid-soluble metabolites in culture supernatants, related to Figure 4E, S8E

Table S9. siRNA sequences used for TLR knockdown experiments, related to Figure 3E

Table S10. Primer sequences used for RT-qPCR, related to Figure 3D–3F, 4G, 4H, S6E, S9A–S9E
