# Supplementary material for: Live and heat-treated Lactiplantibacillus plantarum induce distinct metabolic and immune responses in intestinal epithelial cells
Source: iScience. 2026 Apr 1;29(5):115516. doi: 10.1016/j.isci.2026.115516 (PMC13099361; doi:10.1016/j.isci.2026.115516)
Supplement: Document S1. Figures S1–S9 [file mmc1.pdf]

iScience, Volume *29*

Supplemental information

Live and heat-treated *Lactiplantibacillus plantarum* induce distinct metabolic and immune responses in intestinal epithelial cells

Kaho Matsumoto, Yuta Takada, Yoshiya Imamura, Hina Yoshida, Kazuhiro Sonomura, Mikako Takahashi, Nobuko Moritoki, Tomoko Shindo, Junya Yamamoto, Leonardo Albarracin, Wakako Ikeda-Ohtsubo, Masatoshi Hori, Julio Villena, Fu Namai, Yuji Tsujikawa, Toyoyuki Hashimoto, Keita Nishiyama, and Haruki Kitazawa

Supplemental data

Figures S1−S9

Figure S1. Characterization of the co-culture system, related to **Figure 1**

Figure S2. SEM observation of the interaction between *L. plantarum* and SIECs, related to **Figure 1C**

Figure S3. TEM observation of the interaction between *L. plantarum* and SIECs, related to **Figure 1C**

Figure S4. Transcriptomic, protein, metabolic and oxygen analysis of SIECs following co-culture with *L. plantarum*, related to **Figure 2**

Figure S5. Protein and metabolic profiling of hypoxia-associated responses in SIECs under aerobic and anaerobic conditions, related to **Figure 2**

Figure S6. Transcriptomic and metabolic responses of SIECs following co-culture with heat-treated *L. plantarum*, related to **Figure 3**

Figure S7. Transcriptomic profiles from monocultured *L. plantarum* and co-cultured *L. plantarum* with SIECs and metabolomic profiling of culture supernatants, related to **Figure 4**

Figure S8. Metabolomic profiling of culture supernatants, related to **Figure 4**

Figure S9. Activation of PPARG signaling and lipid metabolism by *L. plantarum*-derived metabolites in SIECs, related to **Figure 4**

Supplemental Figures

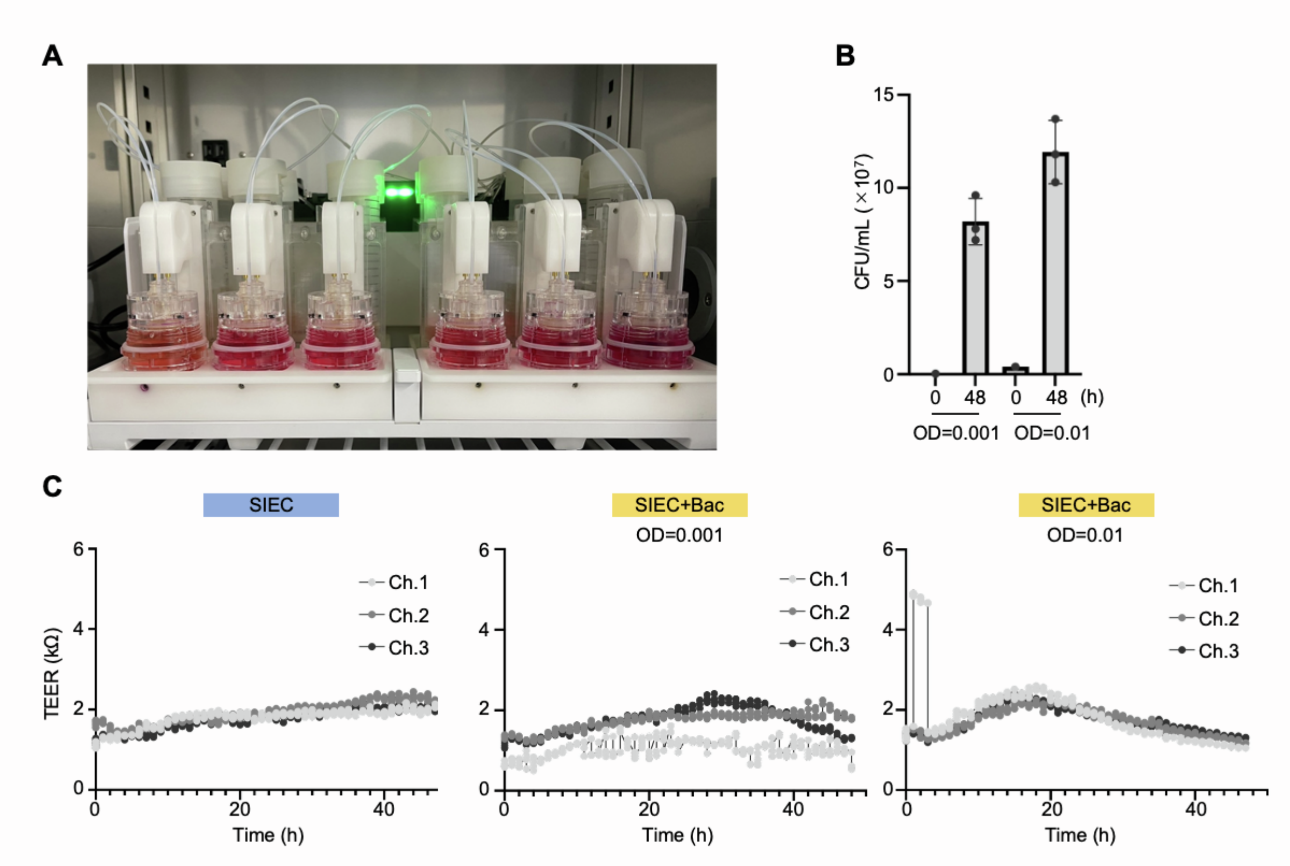

Supplementary Figure 1

Characterization of the co-culture system

(A) Photograph of the co-culture device used for simultaneous culture of *L. plantarum* JCM1149^{T} and swine intestinal epithelial cells (SIECs). **(B)** Colony-forming unit (CFU) counts at 0 and 48 h following inoculation at either 2.8 × 10^5 CFU/mL (OD_{600} = 0.001) or 4.0 × 10^6 CFU/mL (OD_{600} = 0.01). Data are represented as mean ± SD from three biological replicates (*n* = 3). **(C)** Transepithelial electrical resistance (TEER) measurements over time under SIEC (SIEC monoculture) and SIEC+Bac (co-culture with live bacteria) conditions following inoculation at OD_{600} = 0.001 or 0.01. Measurements were performed simultaneously in independent wells (*n* = 3 per condition).

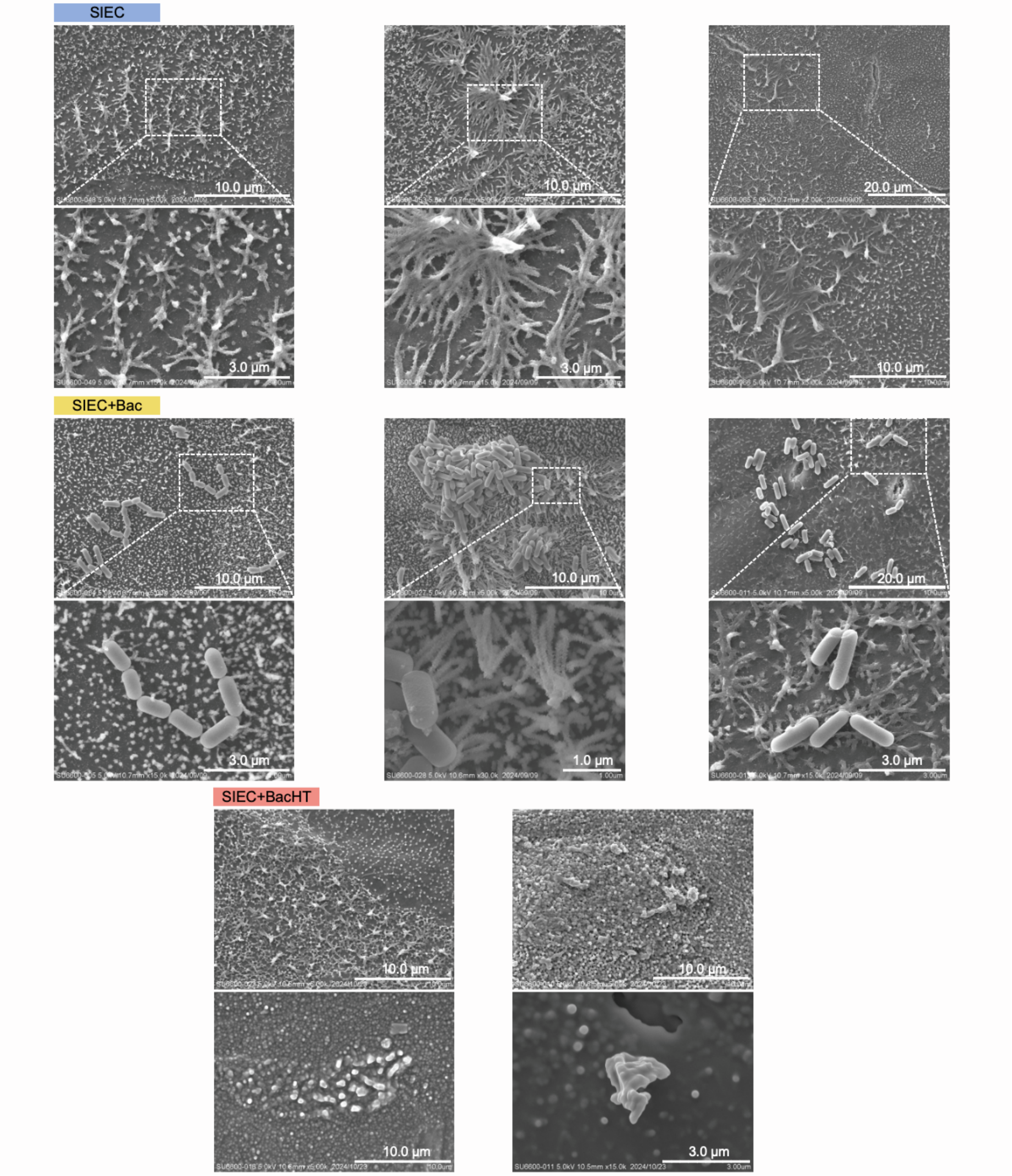

Supplementary Figure 2

SEM observation of the interaction between *L. plantarum* and SIECs

Representative scanning electron microscopy (SEM) images of SIECs under SIEC, SIEC+Bac, and SIEC+BacHT (co-culture with heat-treated bacteria) conditions. For SIEC and SIEC+Bac, lower images are higher-magnification views of the same fields. The analysis was performed once per condition. A total of 30, 28, and 24 randomly selected, non-overlapping fields of view were imaged for SIEC, SIEC+Bac, and SIEC+BacHT, respectively, to ensure representative sampling. Scale bars: 1, 3, 10, or 20 µm.

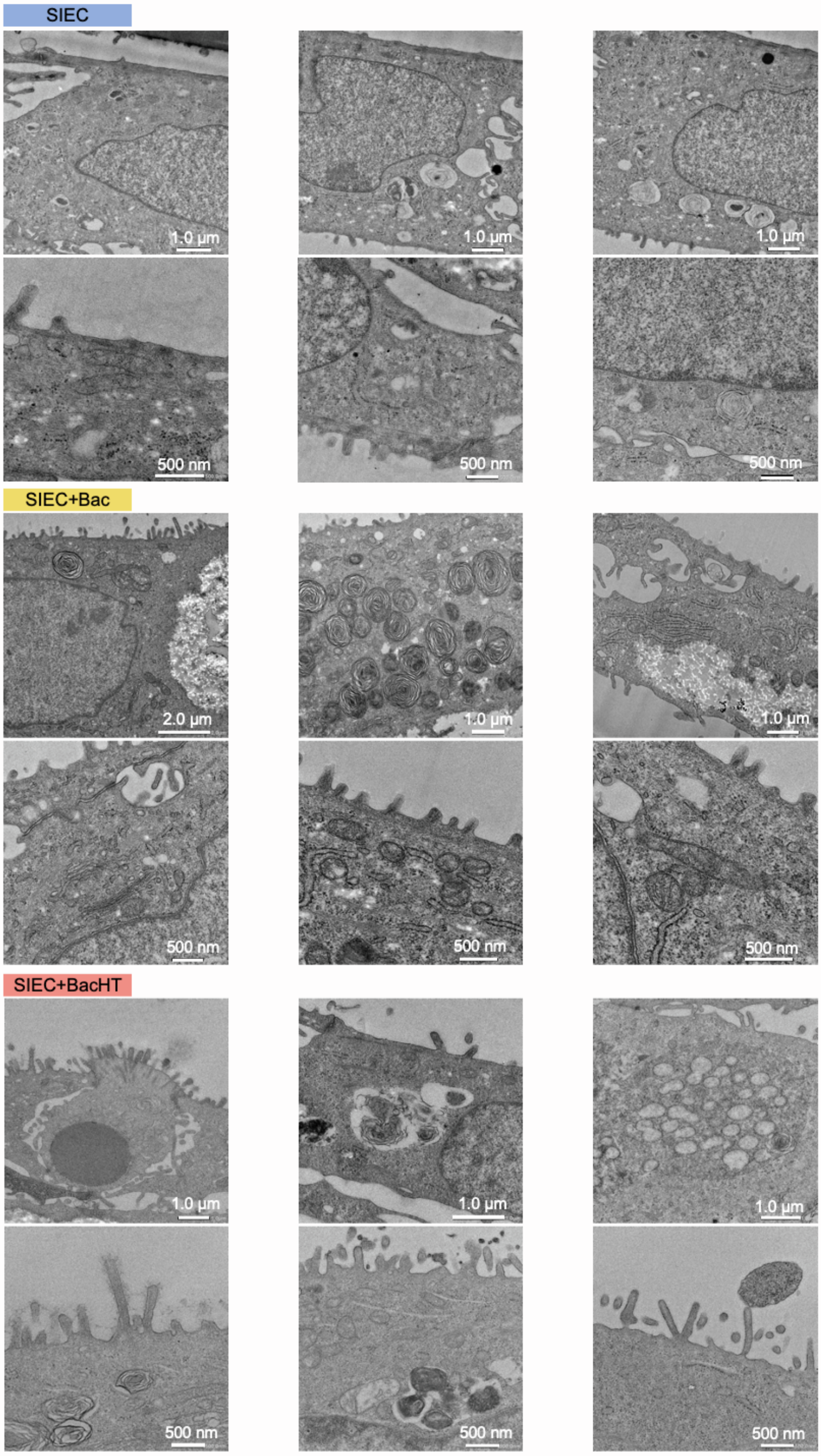

Supplementary Figure 3

TEM observation of the interaction between *L. plantarum* and SIECs

Representative transmission electron microscopy (TEM) images of SIECs under SIEC, SIEC+Bac, and SIEC+BacHT conditions. The analysis was performed once per condition. A total of 19, 18, and 67 randomly selected, non-overlapping fields of view were imaged for SIEC, SIEC+Bac, and SIEC+BacHT, respectively, to ensure representative sampling. Scale bars: 200 nm, 500 nm, 1 µm, or 2 µm.

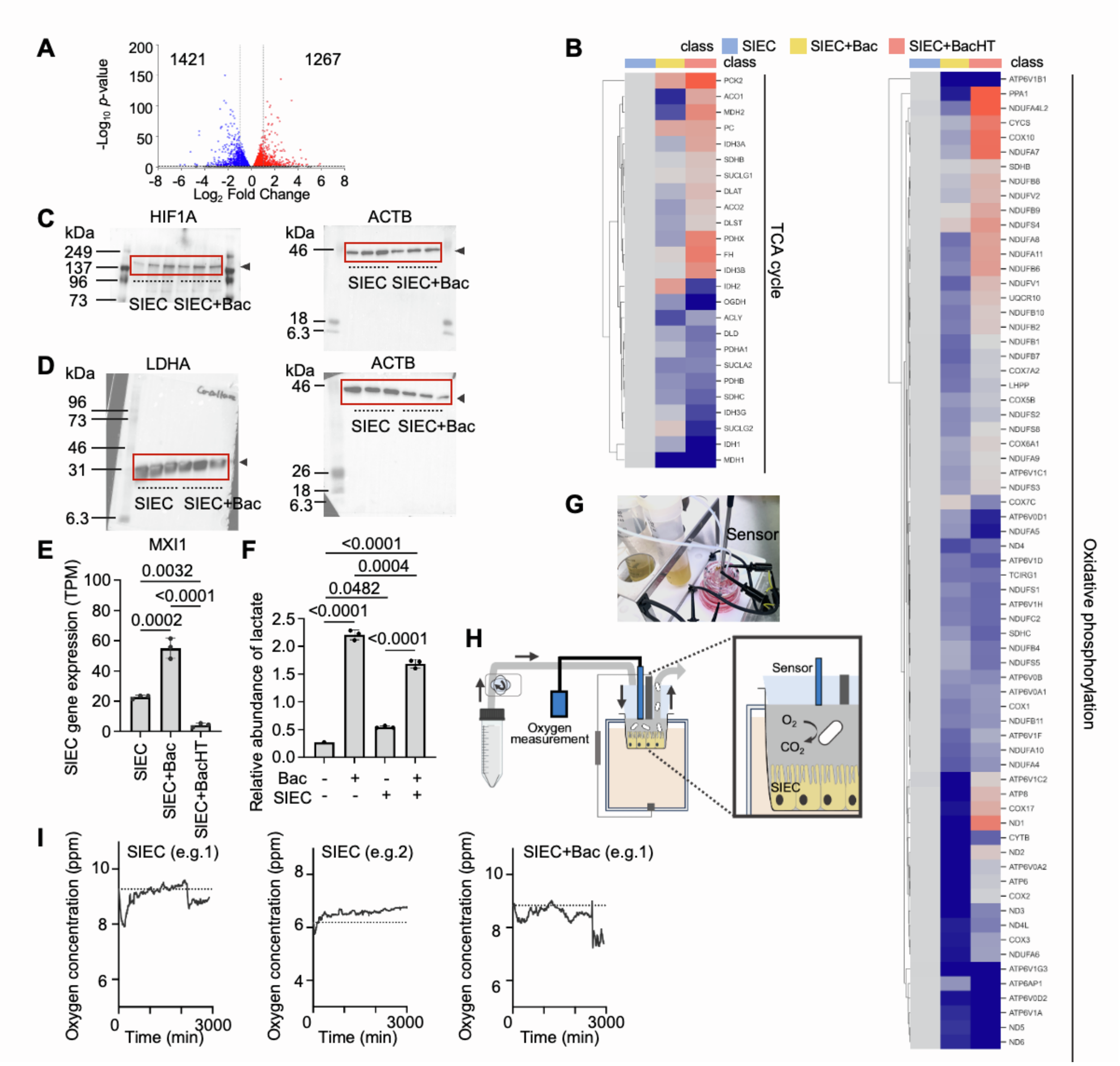

Supplementary Figure 4

Transcriptomic, protein, metabolic and oxygen analysis of SIECs following co-culture with *L. plantarum*.

(A) Volcano plot of differentially expressed genes (DEGs, adjusted $p < 0.001$) comparing SIEC and SIEC+Bac. **(B)** Diagram of the tricarboxylic acid cycle and oxidative phosphorylation pathway showing gene expression changes ($\log_2$ fold change) in SIECs. **(C)** Full, uncropped western blot images of HIF1A and ACTB protein levels in SIECs under the indicated conditions, corresponding to **Fig. 2E**, from three biological replicates (n = 3). **(D)** Full, uncropped western blot images of LDHA and ACTB protein levels in SIECs under the indicated conditions, corresponding to **Fig. 2F,** from three biological replicates (n = 3). **(E)** TPM of *MXI1* in SIECs. Data are represented as mean ± SD from three biological replicates (n = 3). Statistical analysis was performed using one-way ANOVA with Tukey's post-hoc test. **(F)** Peak intensity of lactate measured in culture supernatants. Data are represented as mean ± SD (n = 3). Statistical analysis was performed using one-way ANOVA with Tukey's test. **(G)** Photograph of the oxygen measurement system to measure oxygen concentration under SIEC and SIEC+Bac conditions. **(H)** Schematic diagram of the oxygen measurement system under continuous medium flow. **(I)** Real-time oxygen concentration measured in the culture device under SIEC from three biological replicates (n = 3) and SIEC+Bac conditions from two biological replicates (n = 2). The dotted line on the x-axis indicates the initial oxygen concentration. Data shown here include two datasets and one dataset, respectively, with the remaining dataset shown in **Fig. 2G**.

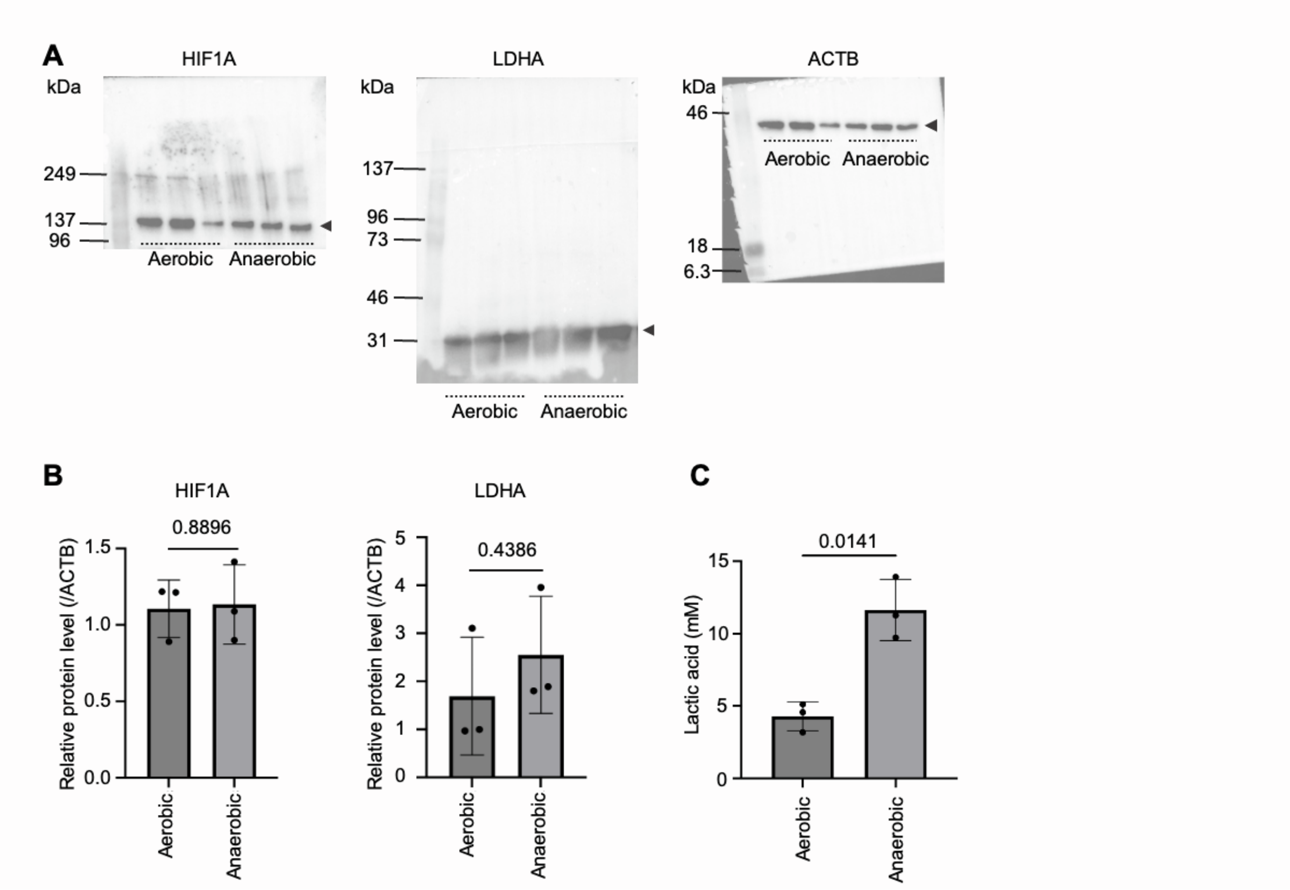

Supplementary Figure 5

Protein and metabolic profiling of hypoxia-associated responses in SIECs under aerobic and anaerobic conditions.

(A) Western blot analysis of HIF1A, LDHA and ACTB protein levels in SIECs under aerobic and anaerobic conditions from three biological replicates (*n* = 3). **(B)** Quantification of band intensities normalized to ACTB and presentation of relative protein levels. Data are represented as mean ± SD from three biological replicates (*n* = 3). Statistical analysis was performed using Welch's t test. **(C)** Peak intensity of lactic acid in culture supernatants. Data are represented as mean ± SD from three biological replicates (*n* = 3). Statistical analysis was performed using Welch's t test.

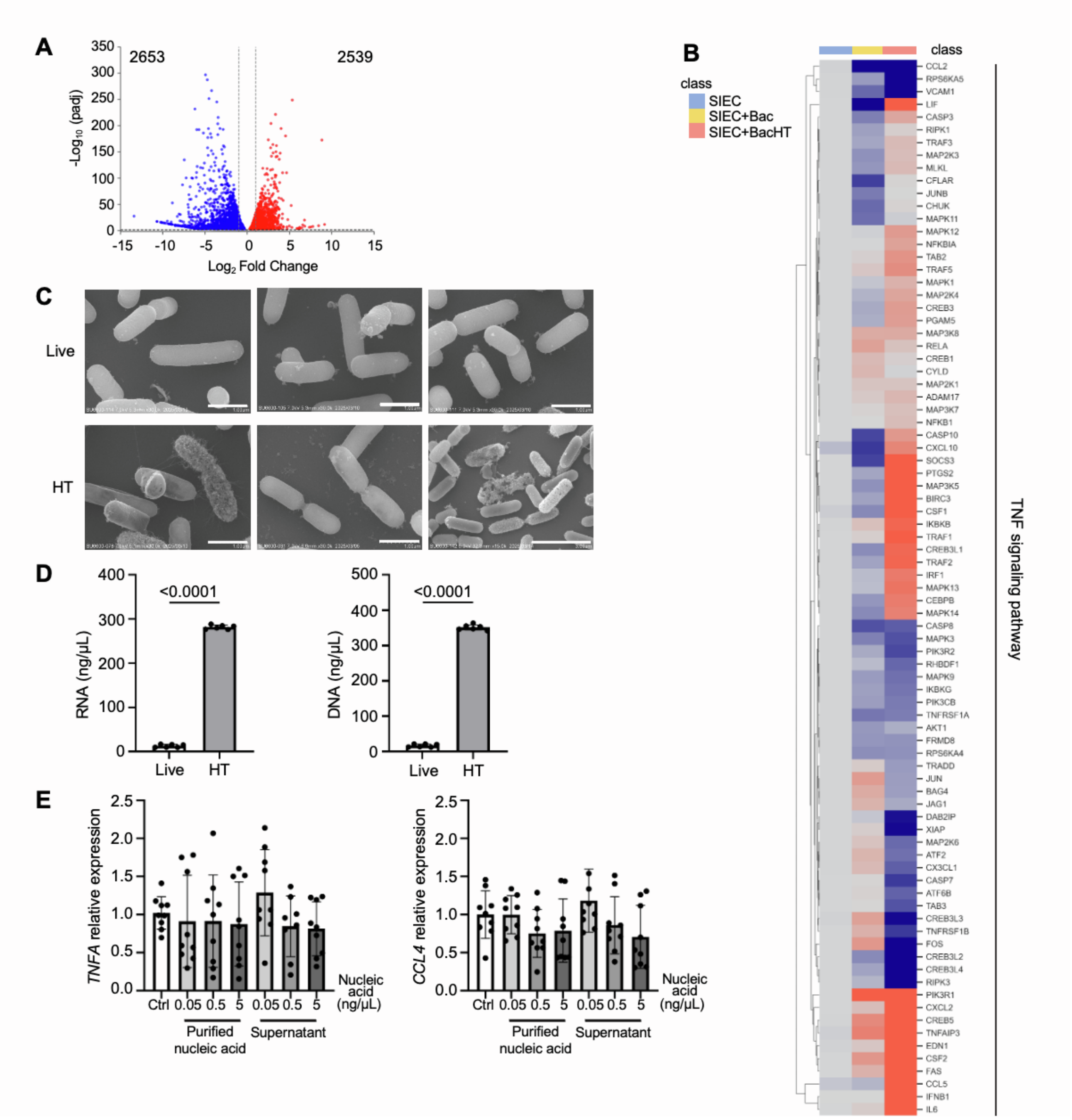

Supplementary Figure 6

Transcriptomic and metabolic responses of SIECs following co-culture with heat-treated *L. plantarum*

(A) Volcano plot illustrating DEGs (adjusted $p < 0.001$) comparing SIEC and SIEC+BacHT. **(B)** Diagram of TNF signaling pathway showing gene expression changes (log_2 fold change) in SIECs. **(C)** Representative SEM images of bacterial cells after heat treatment. The analysis was performed once per condition. A total of 46 and 25 randomly selected, non-overlapping fields of view were imaged for live and heat-treated (HT) bacteria, respectively, to ensure representative sampling. Scale bars: 1 µm and 3 µm. **(D)** Quantification of RNA and DNA concentrations in bacterial supernatants before and after heat treatment (70 °C, 90 min). Data are represented as mean ± SD from three biological replicates ($n = 3$). Statistical analysis was performed using unpaired t-test. **(E)** RT-qPCR analysis of *TNFA* and *CCL4* expression in SIECs stimulated with extracted bacterial nucleic acid mixture or bacterial-derived supernatants. Results expressed as mean ± SD from three biological replicates ($n = 3$), each measured in technical triplicate. Statistical analysis was performed using one-way ANOVA with Dunnett's test.

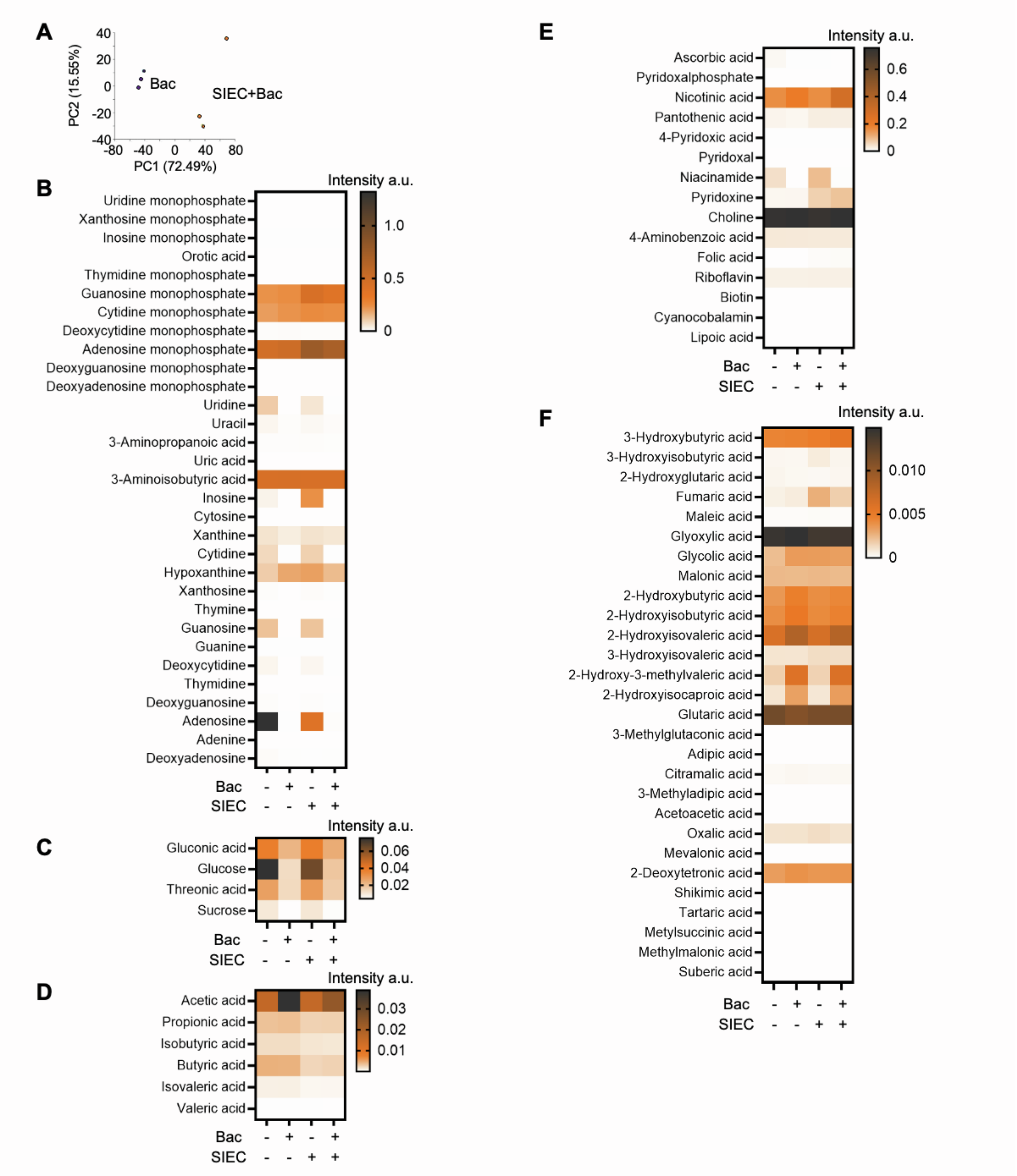

Supplementary Figure 7

Transcriptomic profiles from monocultured *L. plantarum* and co-cultured *L. plantarum* with SIECs and metabolomic profiling of culture supernatants.

(A) Principal component analysis of RNA-sequencing data comparing *L. plantarum* JCM1149^{T} monoculture (Bac) and co-culture (SIEC+Bac) conditions from three biological replicates (n = 3 per condition). **(B–F)** Heatmaps illustrating relative changes in nucleic acids **(B)**, saccharides **(C)**, short-chain fatty acids (SCFAs, **D**), vitamins **(E)**, and organic acids **(F)** in supernatants from Bac and SIEC+Bac conditions, based on mean peak intensities from three independent LC-MS/MS analyses.

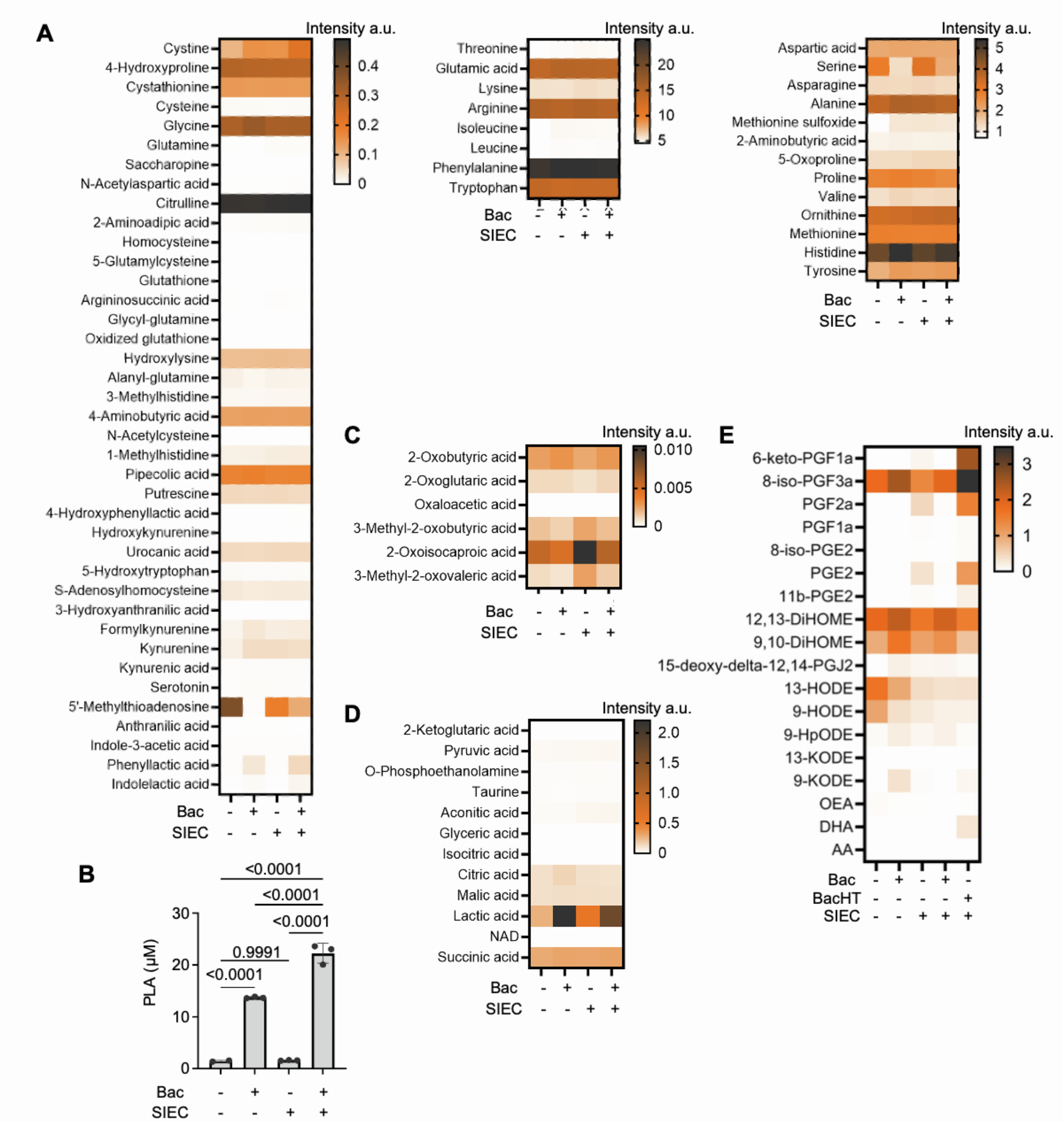

Supplementary Figure 8

Metabolomic profiling of culture supernatants

(A) Heatmap displaying changes in amino acids detected in culture supernatants. **(B)** Peak intensity of phenyllactic acid (PLA) measured in supernatants. Data are represented as mean ± SD from three biological replicates (*n* = 3 per condition). Statistical analysis was performed using one-way ANOVA with Tukey's test. **(C–D)** Heatmaps illustrating changes in α-keto acids **(C)** and miscellaneous metabolites **(D)** detected in supernatants, based on mean peak intensities from three independent LC-MS/MS analyses. **(E)** Heatmap displaying changes in lipid-soluble metabolites detected in supernatants.

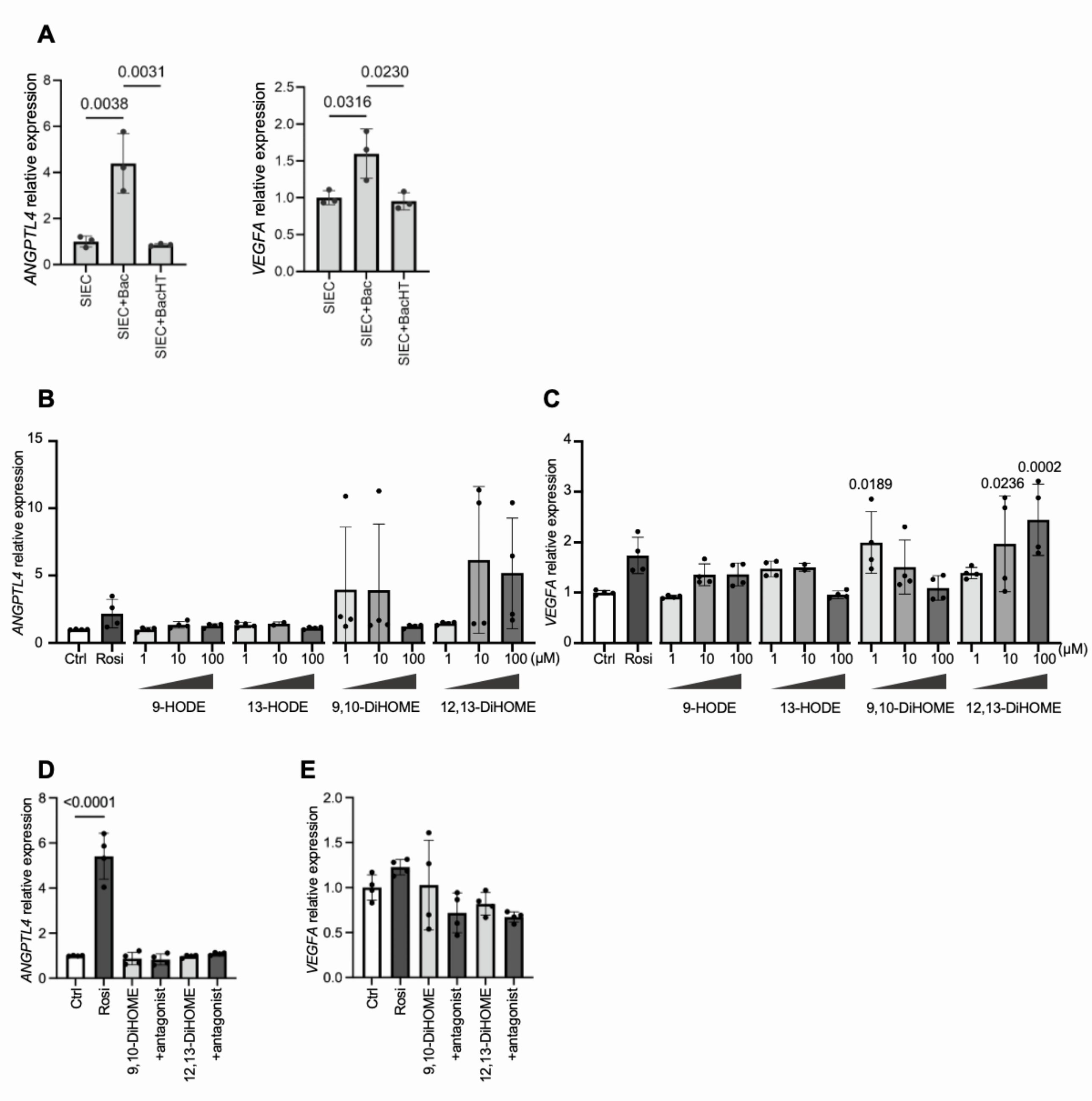

Supplementary Figure 9

Activation of PPARG signaling and lipid metabolism by *L. plantarum*-derived metabolites in SIECs

(A) RT-qPCR analysis of *ANGPTL4* and *VEGFA* expression in SIECs under SIEC, SIEC+Bac, and SIEC+BacHT conditions. Data are represented as mean ± SD from three biological replicates (n = 3), each measured in technical triplicate. Statistical analysis was performed using one-way ANOVA with Tukey's multiple comparisons test. **(B–C)** RT-qPCR analysis of *ANGPTL4* **(B)** and *VEGFA* **(C)** expression in SIECs treated with rosiglitazone (100 μM; positive control) or increasing concentrations (1, 10, 100 μM) of lipid mediators (9-HODE, 13-HODE, 9,10-DiHOME, 12,13-DiHOME). Data are represented as mean ± SD from four biological replicates (n = 4), each measured in technical triplicate. Statistical analysis was performed using one-way ANOVA followed by Dunnett's multiple comparisons test. **(D–E)** RT-qPCR analysis of *ANGPTL4* **(D)** and *VEGFA* **(E)** expression in SIECs treated with rosiglitazone (100 μM; positive control), 9,10-DiHOME (1 μM), or 12,13-DiHOME (10 μM) either alone or in combination with the antagonist (100 μM). Data are represented as mean ± SD from four biological replicates (n = 4), each measured in technical triplicate. Statistical analysis was performed using one-way ANOVA followed by Dunnett's multiple comparisons test.

REFERENCES

[S1] Shimazu, T., Villena, J., Tohno, M., Fujie, H., Hosoya, S., Shimosato, T., Aso, H., Suda, Y., Kawai, Y., Saito, T., et al. (2012). Immunobiotic Lactobacillus jensenii Elicits Anti-Inflammatory Activity in Porcine Intestinal Epithelial Cells by Modulating Negative Regulators of the Toll-Like Receptor Signaling Pathway. Infection and Immunity *80*, 276–88. https://doi.org/10.1128/iai.05729-11.

[S2] Zheng, Z., Lyu, W., Hong, Q., Yang, H., Li, Y., Zhao, S., Ren, Y., and Xiao, Y. (2023). Phylogenetic and expression analysis of the angiopoietin-like gene family and their role in lipid metabolism in pigs. Anim Biosci *36*, 1517–29. https://doi.org/10.5713/ab.23.0057.

[S3] Zhang, J., Peng, X., Yuan, A., Xie, Y., Yang, Q., and Xue, L. (2017). Peroxisome proliferator-activated receptor γ mediates porcine placental angiogenesis through hypoxia inducible factor-, vascular endothelial growth factor- and angiopoietin-mediated signaling. Mol Med Rep *16*, 2636–44. https://doi.org/10.3892/mmr.2017.6903.

[S4] Kobayashi, H., Albarracin, L., Sato, N., Kanmani, P., Kober, A.K., Ikeda-Ohtsubo, W., Suda, Y., Nochi, T., Aso, H., Makino, S., et al. (2016). Modulation of porcine intestinal epitheliocytes immunetranscriptome response by *Lactobacillus jensenii* TL2937. Benef Microbes *7*, 769–82. https://doi.org/10.3920/bm2016.0095.
